# Genome-wide analysis of autophagy-associated genes in foxtail millet (*Setaria italica* L.) and characterization of the function of *SiATG8a* in conferring tolerance to nitrogen starvation in rice

**DOI:** 10.1186/s12864-016-3113-4

**Published:** 2016-10-12

**Authors:** Weiwei Li, Ming Chen, Erhui Wang, Liqin Hu, Malcolm J. Hawkesford, Li Zhong, Zhu Chen, Zhaoshi Xu, Liancheng Li, Yongbin Zhou, Changhong Guo, Youzhi Ma

**Affiliations:** 1 Key Laboratory of Molecular Cytogenetics and Genetic Breeding of Heilongjiang Province, College of Life Science and Technology, Harbin Normal University, Harbin, Heilongjiang 150025 China; 2 Institute of Crop Sciences, Chinese Academy of Agricultural Sciences, National Key Facility for Crop Gene Resources and Genetic Improvement, Key Laboratory of Biology and Genetic Improvement of Triticeae Crops, Ministry of Agriculture, Beijing, 100081 China; 3 College of Life Sciences, Northwest A&F University, Yangling, Shanxi 712100 China; 4 Plant Biology and Crop Science Department, Rothamsted Research, Harpenden, Hertfordshire AL5 2JQ UK; 5 Department of Chemistry, University of Science and Technology of China, Hefei, Anhui 230000 China

**Keywords:** Foxtail millet, Autophagy-associated genes, Expression pattern, Functional identification, Nitrogen starvation

## Abstract

**Background:**

Autophagy is a cellular degradation process that is highly evolutionarily-conserved in yeast, plants, and animals. In plants, autophagy plays important roles in regulating intracellular degradation and recycling of amino acids in response to nutrient starvation, senescence, and other environmental stresses. Foxtail millet (*Setaria italica*) has strong resistance to stresses and has been proposed as an ideal material for use in the study of the physiological mechanisms of abiotic stress tolerance in plants. Although the genome sequence of foxtail millet (*Setaria italica*) is available, the characteristics and functions of abiotic stress-related genes remain largely unknown for this species.

**Results:**

A total of 37 putative *ATG* (autophagy-associated genes) genes in the foxtail millet genome were identified. Gene duplication analysis revealed that both segmental and tandem duplication events have played significant roles in the expansion of the *ATG* gene family in foxtail millet. Comparative synteny mapping between the genomes of foxtail millet and rice suggested that the *ATG* genes in both species have common ancestors, as their *ATG* genes were primarily located in similar syntenic regions. Gene expression analysis revealed the induced expression of 31 *SiATG* genes by one or more phytohormone treatments, 26 *SiATG* genes by drought, salt and cold, 24 *SiATG* genes by darkness and 25 *SiATG* genes by nitrogen starvation. Results of qRT-PCR showing that among 37 *SiATG* genes, the expression level of *SiATG8a* was the highest after nitrogen starvation treatment 24 h, suggesting its potential role in tolerance to nutrient starvation. Moreover, the heterologous expression of *SiATG8a* in rice improved nitrogen starvation tolerance. Compared to wild type rice, the transgenic rice performed better and had higher aboveground total nitrogen content when the plants were grown under nitrogen starvation conditions.

**Conclusions:**

Our results deepen understanding about the characteristics and functions of *ATG* genes in foxtail millet and also identify promising new genetic resources that should be of use in future efforts to develop varieties of foxtail millet and other crop species that have resistance to nitrogen deficiency stress.

**Electronic supplementary material:**

The online version of this article (doi:10.1186/s12864-016-3113-4) contains supplementary material, which is available to authorized users.

## Background

The yield and quality of crops are adversely influenced under adverse environmental conditions, such as high salt, drought, and nutrient starvation. In order to survive, plants have evolved various capacities that enable them to resist or adapt to environmental changes. Autophagy is a highly evolutionarily-conserved cellular degradation process that is induced by nutrient starvation and it is essential for recycling of the cellular cytoplasmic contents and the breakdown of damaged proteins [1, 2]. In autophagy, the macromolecules or organelles are sequestered into a double-membraned vesicle termed an autophagosome. The content of autophagosome ranges from damaged proteins to obsolete organelles. This content is finally delivered into a vacuole (in yeast and plants) or a lysosome (animals) for degradation and nutrient recycling during periods of stress and starvation [1, 2]. Autophagy maintains cellular homeostasis during stressful conditions and plays important roles in various cellular processes. Therefore, the molecular and physiological mechanisms of plant autophagy are attracting more and more research attention.

Identification of genes that participate in autophagy should establish a foundation for furthering our understanding of the molecular mechanism and the potential roles of autophagy in different physiological processes. So far, most of our knowledge about autophagy comes from studies of yeast. In yeast, the number of *ATG* genes currently stands at 41 [3–7]. A group of autophagy-defective mutants have been isolated and used to study the autophagy process in yeast [8–12]. The studies of these yeast mutants have revealed the molecular mechanisms underlying autophagy [13]. The plant and mammalian homologues of the yeast *ATG* genes have been found, and these show conservation of the core ATG mechanism during evolution. Recently, some *ATG* homologues have been found and functionally analyzed in the genomes of *Arabidopsis*, rice, and maize [14–17]. Through genome sequencing, a total of 33 *ATG* homologues are known to exist in the rice (*Oryza sativa*) genome, and more than 30 *ATG* genes have been identified in *Arabidopsis thaliana*, tobacco, maize [16–19]. Autophagy is involved in a variety of processes in plant metabolism and development, including apoptotic processes, senescence, nutrient starvation, vacuole biogenesis, and seed development. Autophagy is related to responses to biotic and abiotic stresses such as viral infection and oxidative, salt, and drought stress [1, 20–22]. Under normal conditions, autophagy is kept at a low level, but it can be activated by nutrient starvation, which is known to induce the expression of some *ATG* genes [23]. The rice mutant *Osatg7-1* has reduced biomass production and nitrogen use efficiency compared with wild type plants [24]. Under nitrogen starvation, the maize *atg12* mutant shows enhanced leaf senescence and stunted ear development. Nitrogen partitioning studies revealed that seed yield and the nitrogen-harvest index were significantly decreased in the *atg12* mutant [17]. Some *Arabidopsis* autophagy-defective mutants are also known to be particularly sensitive to nutrient starvation. Transgenic *Arabidopsis* plants of an ATG-knockdown line, RNAi-*ATG18a*, have been shown to be more sensitive than wild type plants to nutrient-limiting conditions [25]. The *Arabidopsis atg* mutants, such as *atg4a4b-1*, *atg5-1, atg7-1, atg9-1* and *atg10-1* exhibited abnormal phenotypes (chlorosis, decreased bolting time, and early senescence) under nutrient starvation [21, 22, 25–29]. ATG8, one of the conserved autophagy proteins, is a lipid-conjugated ubiquitin-like protein that functions as a scaffold for membrane expansion during autophagosome formation [30, 31]. In contrast to the single *ATG8* gene present in the genome of budding yeast, there are nine and six *ATG8* orthologues present in the *Arabidopsis* and rice genomes, respectively [16, 32]. In plant cells, AtAtg8 synthetic substrates can be efficiently incorporated into autophagosomes, and this processing is known to require AtAtg4s. Synthetic AtAtg8 substrate could be processed efficiently in an AtAtg4-dependent manner. These results indicate that, in vivo*,* the synthetic AtAtg8 substrate is used efficiently in the biogenesis of autophagosomes [33]. Under nitrogen starvation, the expression and transcription patterns of *OsATG8* homologues were different in various tissues, indicating that the functions among homologues maybe different [16]. In plants, *ATG8* genes are known to function efficiently in cellular processes in young, non-senescing tissues, under both favorable growth conditions and under starvation stress [34]. *TdATG8,* from wild emmer wheat, may play a role in a drought tolerance mechanism and *TdATG8* is known to be a positive regulator of osmotic and drought stress responses [35]. Expression of *AtATG8* in *Arabidopsis* increased plant sensitivity to mild salt and osmotic stress [36]. Heterologous expression of *GmATG8c* in *Arabidopsis* conferred tolerance to nitrogen deficiency and increased yields [37]. To date, plant autophagy research has been concentrated on model plant species, few studies have explored the functions of ATG-like genes in non-model crop species.

Foxtail millet (*Setaria italica* L.) is a member of the Poaceae grass family and is an ancient crop in China. Foxtail millet is an important food and fodder crop in arid regions because of its characteristics such as resistance to drought and nutrition deficiency stress. Published plant genome sequences have facilitated research on foxtail millet [38–40]. However, there have been few articles reporting studies related to stress-related gene function in foxtail millet so far. In this study, 37 *ATG* genes in the foxtail millet genome (internally annotated as *Setaria italica* autophagy- associated genes; *SiATG*s) were identified. We determined their chromosomal locations, predicted the protein structures and analyzed the expression patterns of these 37 *SiATG* genes with qRT-PCR. The results showed that among *SiATG* genes the expression level of *SiATG8a* was the highest after low nitrogen treatment 24 h and overexpression of *SiATG8a* enhanced the tolerance of transgenic rice to nitrogen starvation stress. Our results deepen understanding about the characteristics and functions of *ATG* genes in foxtail millet and also identify promising new genetic resources for foxtail millet and other crop species that have resistance to nitrogen deficiency stress.

## Results

### Bioinformatics analysis of 37 *SiATG* genes: identification and chromosomal distribution

A total of 37 putative *ATG* genes were identified in foxtail millet via genome-wide analysis (Additional file 1: Table S1). Their domains were further confirmed with the Pfam database (http://pfam.xfam.org/). The lengths of the amino acid sequences, the predicted molecular weights, and the predicted isoelectric points (pI) (Additional file 1: Table S1 and S2) differed greatly among the 37 SiATG proteins. The coding sequences, genomic sequences, protein sequences and nucleotide sequences upstream of the initiation codon for transcription for the 37 Si*ATGs* were all downloaded from (www.phytozome.net/). The deduced SiATG protein sequences ranged from 91 amino acids (SiATG12) to 2474 amino acids (SiTOR), and the corresponding molecular weights varied from 10 to 278 kDa. The predicted isoelectric points varied from 4.53 (SiATG3a) to 9.51 (SiATI12a). According to information on the phytozome website, ten proteins were found to be splice variants of primary transcripts encoding autophagy-associated proteins: SiATG1, SiATG5, SiATG8b, SiATG8d, SiATG10, SiATG16, SiTOR, SiVPS15, SiVPS34, and SiNBR1 (Additional file 1: Table S1).

**Fig. 1:**
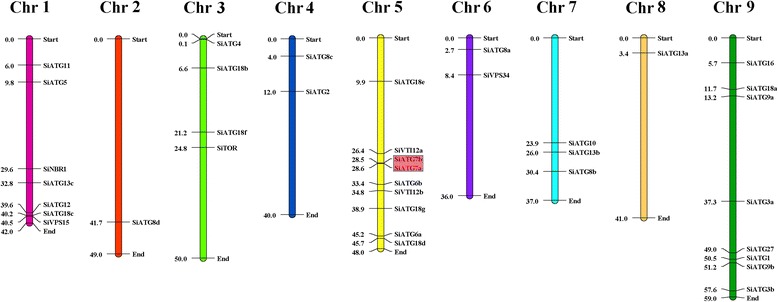
Distribution of 37 *SiATG* genes onto the nine foxtail millet chromosomes. Localization of the foxtail millet *ATG* genes on the foxtail millet chromosomes. Tandem duplicated genes on a particular chromosome are indicated with colored boxes. Chromosomal distances are given in Mb

Foxtail millet has nine chromosomes, ranging in size from 35.9 Mb (chromosome 6) to 58.9 Mb (chromosome 9). The physical map positions of the 37 *SiATG* genes in the nine chromosomes of foxtail millet are presented in Fig. 1. The specific location of each *SiATG* gene on the chromosomes is given in Additional file 1: Table S1. Subcellular localization predictions revealed that most of the SiATG proteins were predicted to be localized to the cytoplasm or the nucleus (Additional file 1: Table S2).

### Phylogenetic analysis of foxtail millet ATGs with ATGs of other plant species, and gene structure analysis

**Fig. 2:**
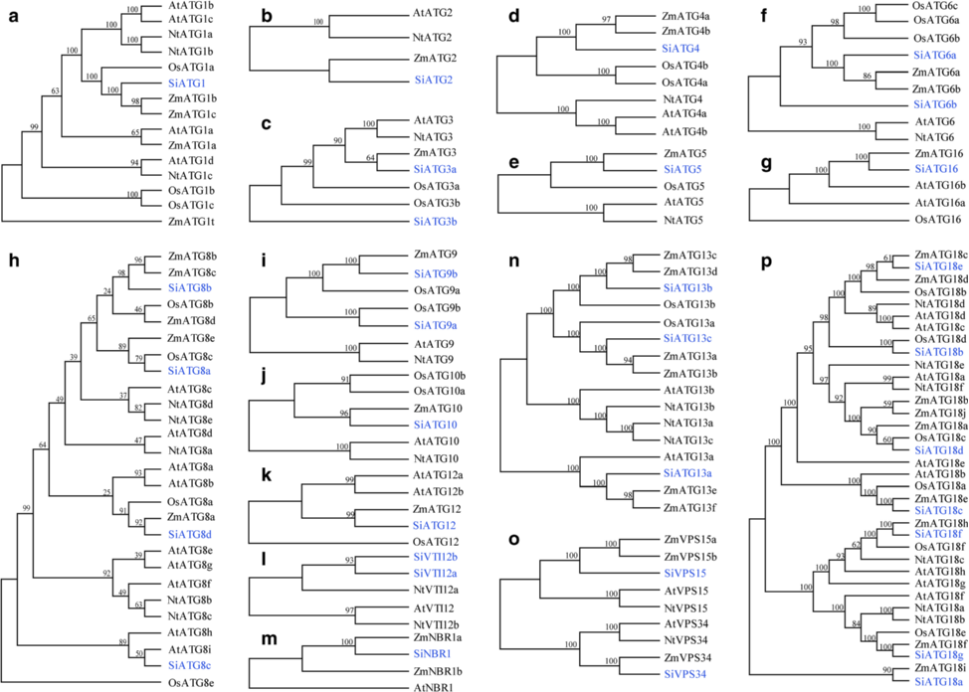
Phylogenetic analysis of foxtail millet and other plant ATGs. Phylogenetic relationships of ATGs from *S. italica* (Si) with those of *N. tabacum* (Nt), *A. thaliana* (At), *O. sativa* (Os), and *Z. mays* (Zm). The tree was constructed from an analysis conducted with MEGA 5.1 software using a maximum likelihood method. The names in blue color are the foxtail millet ATGs

In recent years, the autophagy-associated genes in multiple plant species have been identified. We chose several representational plants for analysis, including *Arabidopsis thaliana* (At), *Oryza sativa* (Os), *Nicotiana tabacum* (Nt) and *Zea mays* (Zm) and constructed the phylogenetic trees of the autophagy-associated genes in these species, in addition to *Setaria italica* (Si). The multiple sequence alignments of the ATG protein sequences were conducted with the ClustalX 1.81 program, using the default multiple alignment parameters. The unrooted phylogenetic trees were constructed using MEGA5.0 software by maximum likelihood method with 1000 bootstrap replicates to compare the evolutionary relationships. As shown in Fig. 2, the phylogenetic analysis suggested that each SiATG protein sequence was highly similar to their homologues in other plant species. Since a good number of the internal branches were observed to have high bootstrap values, it clearly shows the derivation of statistically reliable pairs of possible orthologues proteins sharing similar functions from a common ancestor. The phylogenetic tree also revealed that majority of foxtail millet SiATG families distribution predominates with species bias, they are more closely related to those in grass species (*O. sativa* and *Z. mays*,). These results are consistent with the present understanding of plant evolutionary history [41]. Close association of SiATG families with their counter-parts in other plants, expressions or functions for some of which have reported, may be an implication of sequence conservation and evidence to their similar biological roles. Being a rational systematic approach, such phylogeny-based function prediction has been applied for prediction of stress-responsive proteins in other species like rice, *Arabidopsis* and maize.

As with other plants, some foxtail millet ATG members are encoded by small gene families, implying redundancy and subfunctionalization (ATG8, ATG13, ATG18). Other foxtail millet ATG members are encoded by a single gene (ATG1, ATG2, ATG4, ATG5, ATG10, ATG11, ATG12, ATG16,ATG27, TOR, VPS15, VPS34,and NBR1), making them attractive research materials for reverse genetics studies [17].

**Fig. 3:**
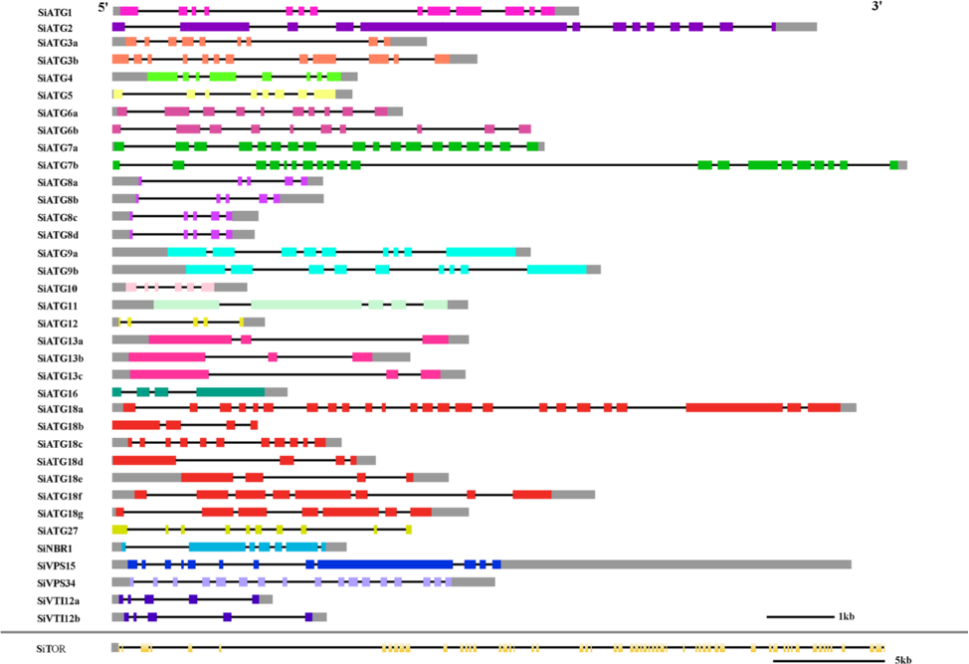
Exon–intron structure of the foxtail millet ATG genes. The exon-intron structures of the *SiATG* genes were determined by comparing the coding sequences and the corresponding genomic sequences using the Gene Structure Display Server (GSDS) program. Gray and colored boxes represent untranslated upstream / downstream regions and coding regions, respectively. Lines indicate introns

**Fig. 4:**
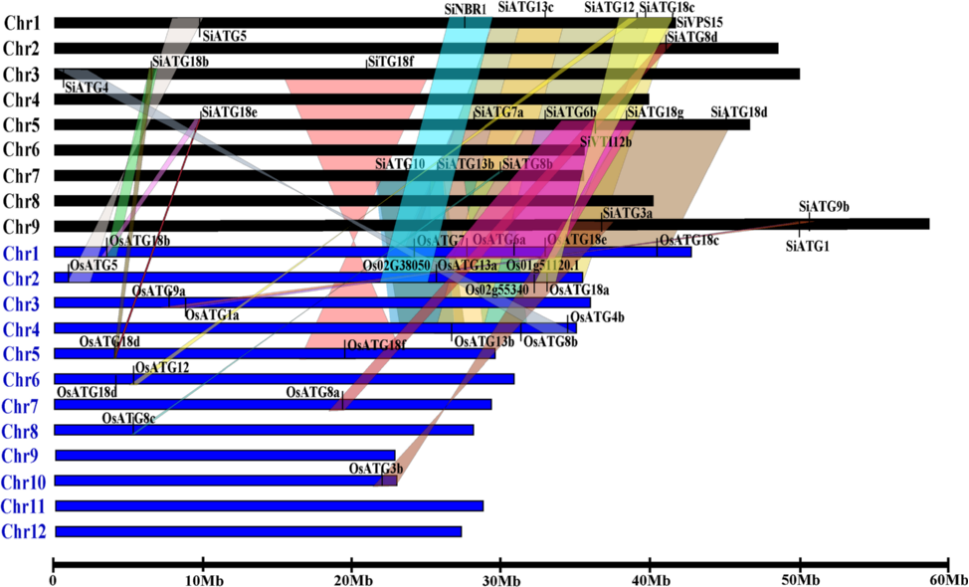
Synteny analysis of the *ATG* genes between foxtail millet and rice. Foxtail millet and rice chromosomes are depicted as black and blue bars, respectively. Foxtail millet and rice *ATG* genes are indicated by vertical black lines. Colored bars denote syntenic regions between foxtail millet and rice chromosomes. A twisted colored bar indicates that syntenic regions have opposite orientations

Exon-intron structural divergence within gene families can play pivotal roles in the evolution of multi-gene families [42]. Exon-intron structural analysis revealed that members of some *ATG* subfamilies have the same number of exons and similar exon-intron structures. Examples of this scenario include *SiATG9a/ SiATG9b* and *SiATG8a/ SiATG8b/ SiATG8c/ SiATG8d* (Fig. 3). To further explore the origins and evolutionary processes of the foxtail millet *ATG* genes, a comparative synteny map between the foxtail millet and rice genomes was constructed (Fig. 4). Rice is an important model plant species, and the genome-wide identification and expression analysis of the rice *ATG* genes has been reported [16]. Thus, through comparative genomics analysis, the probable functions of the foxtail millet *ATG* genes can be inferred. Synteny analysis indicated that 59 % (22/37) of the *SiATG* genes showed synteny with their orthologues in the rice genome, and there were 28 pairs that unambiguously exist in both the foxtail millet and rice genomes (e.g.,*SiATG3a/OsATG3b*, *SiATG5/OsATG5,* and *SiATG7a/OsATG7*). Although there is a close evolutionary relationship between foxtail millet and rice, some *SiATG* genes were not mapped onto any colinear blocks with rice (e.g., *SiATG8a* and *SiATG8c*). This result suggests that the chromosomes of foxtail millet have undergone extensive rearrangements during genomic evolution.

### Duplication and divergence rates of the *SiATG* genes

The distribution of the 37 *SiATG* genes on the nine foxtail millet chromosomes was asymmetric. Only one pair of *SiATG* genes (*SiATG7a /SiATG7b*) occurred as tandem repeats. Another 10 genes (*SiATG13b/ SiATG13c*, *SiATG9a/ SiATG9b*, *SiATG6a/ SiATG6b*, *SiATG8a/ SiATG8b*, *SiATG18b/ SiATG18e*) occurred as five pairs of segmentally-duplicated genome regions (Table 1). The Ka/Ks ratios of these five segmental duplicated gene pairs ranged from 0.028 to 0.420, with an average of 0.210, whereas the Ka/Ks ratio of the aforementioned tandem duplication gene pair was 0.340 (Table 1). One tandem repeat and five segmental duplicated genome regions belonged to paralogous pairs, and among six paralogous pairs, four pairs showed Ks values from 0.932 to 1.674, suggesting that these duplications might have occurred 71.7 million year ago (MYA) in the foxtail millet genome (Table 1). Based on the Ka/Ks ratios, the dates of the four paralogous pair duplication events were calculated to have occurred from between 71.72 to 128.76 MYA (Table 1). The Ks values of the *SiATG7a/ SiATG7b* and *SiATG6a/ SiATG6b* paralogous pairs were 0.0679 and 0.3522, respectively, suggesting that the dates of these two paralogous pair duplication events might have occurred about 5.22 and 27 MYA, respectively. We also calculated the Ka/Ks ratios of the duplicated genes between orthologous gene pairs of foxtail millet and maize (31 pairs) (Additional file 1: Table S4), rice (28 pairs) (Additional file 1: Table S5), and sorghum (29 pairs) (Additional file 1: Table S6).

**Table 1:** Ka/Ks analysis and estimation of the absolute dates for the duplication events between the duplicated SiATG homologues

Duplicated pair	Ks	Ka	Ka/Ks	Duplicate type	Purifying selection	Date (million years)
SiATG6a/SiATG6b	0.3522	0.1482	0.4208	Segmental	YES	27.09
SiATG8a/SiATG8b	0.9323	0.0265	0.0284	Segmental	YES	71.72
SiATG9a/SiATG9b	1.0214	0.2045	0.2002	Segmental	YES	78.57
SiATG13b/SiATG13c	1.0042	0.213	0.2121	Segmental	YES	77.25
SiATG18b/SiATG18e	1.6740	0.3135	0.1873	Segmental	YES	128.76
SiATG7a/SiATG7b	0.0679	0.0231	0.3402	Tandem	YES	5.22

Among the orthologous gene pairs of *SiATG* with other grass species, the highest average Ka/Ks value was between sorghum and foxtail millet (0.2061) and the lowest average Ka/Ks value were between maize and foxtail millet (0.1826). The rate of Ks of *ATG* genes between rice and foxtail millet was relatively higher than between sorghum and maize, with the divergence between foxtail millet and rice probably occurring between 36-95 MYA (Additional file 1: Tables S4–S6). Conversely, the Ka/Ks ratio of the *ATG* gene pairs between foxtail millet and maize (Ka/Ks = 0.1826) was lower than that of sorghum and foxtail millet (average Ka/Ks = 0.2061) and that of foxtail millet and rice (Ka/Ks = 0.1834). These results suggest that intense purifying selection between foxtail millet and maize was more significant than that of sorghum and foxtail millet, and foxtail millet and that of rice. The divergence time between foxtail millet and maize was at the latest17–82 MYA.

### Differential expression profiles of the foxtail millet *ATG* genes under phytohormone, abiotic stress, and nitrogen and carbon starvation treatments

**Fig. 5:**
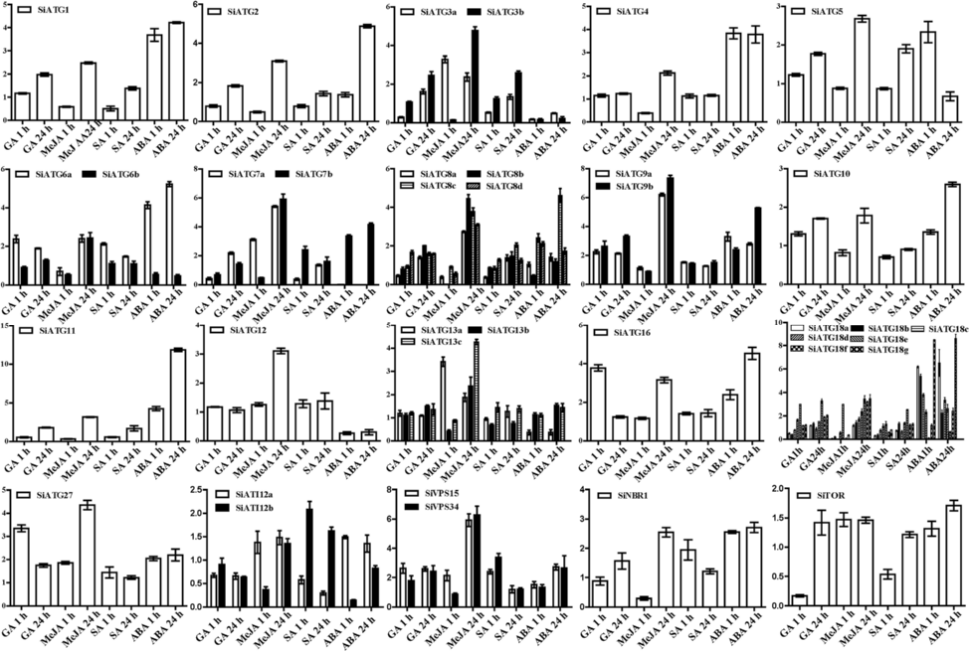
Expression profiles of 37 *SiATG* genes in response to treatment with various hormones. Relative expression levels of each *ATG* in foxtail millet seedlings under various hormones were normalized to Actin mRNA (AF288226.1). 3-week-old seedlings treated with 5μM gibberellic acid (GA), 5μM methyl jasmonate (MeJA), 5μM salicylic acid (SA) and 5μM abscisic acid (ABA) for 1 h and 24 h, respectively. The expression level of each *ATG* was calculated and compared with those of seedlings under normal growth conditions

**Fig. 6:**
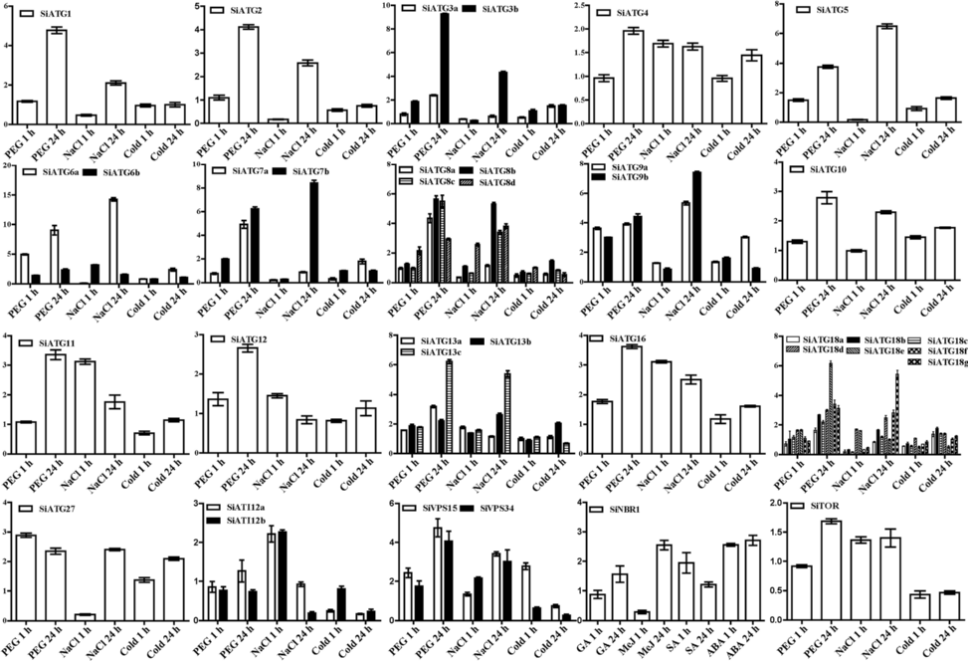
Expression profiles of 37 *SiATG* genes in response to various abiotic stresses. The relative expression levels of each *ATG* gene in foxtail millet seedlings under drought, salt, and cold stresses were normalized to Actin mRNA (AF288226.1). 3-week-old seedlings treated with high salt (100mM NaCl), drought (6 % PEG 6000) stresses, and cold stress (4 °C) for two time durations (1 h and 24 h). The expression level of each *ATG* gene was calculated and compared with that of seedlings grown under normal growth conditions

**Fig. 7:**
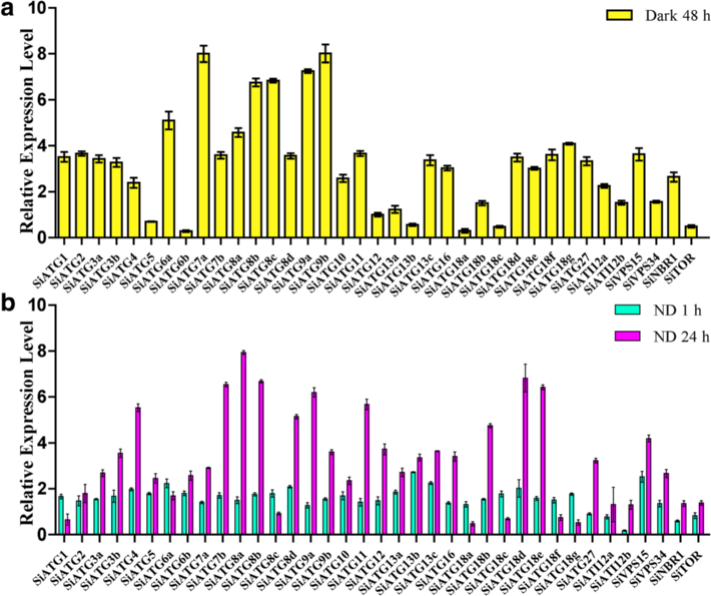
Expression analysis of 37 *SiATG* genes in response to nitrogen deficiency and darkness treatment using qRT-PCR. **a** 3-week old foxtail millet seedlings were kept in darkness for 48 h. **b** foxtail millet seeds were surfaced sterilized, and then seeds were germinated on MS and cultured for 14 days. On day 15, some seedlings were cultured on nitrogen-deficient liquid medium for 1 h or 24 h. The relative expression levels of each *ATG* gene in seedlings grown under darkness and nitrogen starvation were normalized to the Actin mRNA level (AF288226.1). The expression level of each *ATG* gene was calculated and compared with that in seedlings under normal growth conditions. ND: N deficient

To investigate the tissue-specific expression of the 37 *SiATG* genes in foxtail millet, total RNA from roots, stems, and leaves were prepared and analyzed by qRT-PCR. As shown in Additional file 1: Figure S1, the expression of these *SiATG* genes was very low under normal conditions. The expression levels of the *SiATGs* in response to various treatments were quantified with the goal of identifying some key *ATGs* involved in responses to stresses (Figs. 5, 6 and 7 and Additional file 1: Table S8). All of the *SiATG* genes showed differential expression profiles in response to the various phytohormone treatments (Fig. 5). In particular, the expression levels of *SiATG7a* and *SiATG7b* increased significantly under MeJA (methyl jasmonate) treatment. Often, multiple members of the same subfamilies had similar expression patterns, for example: *SiATG9a* and *SiATG9b*.

To investigate the effect of different abiotic stresses treatments on the expression the foxtail millet *ATG* genes, we used qRT-PCR to monitor the expression patterns of the 37*ATG* genes in plants grown under drought, salt, and cold treatment (both 1 h and 24 h duration treatments). As shown in Fig. 6, the expression of most of the *SiATG* genes was either induced or suppressed as a result of these abiotic stress treatments. Although it was the case that the expression of most *SiATG* homologues was altered during the early stages of drought treatment, the expression of all 37 *SiATG* genes had clearly increased during the later stages of treatment. The expression of most of the *SiATG* genes was down-regulated during the early cold stress treatment, although the expression of seven genes was increased slightly (e.g.,*SiATG10*, *SiATG16,* and *SiATG27*).

To study the expression patterns of the 37 *SiATG* genes in response to carbon starvation stress, foxtail millet plants were kept in darkness for 48 h. As shown in Fig. 7a, seven *SiATG* genes (*SiATG6a*, *SiATG7a*, *SiATG8a*, *SiATG8b*, *SiATG8c*, *SiATG9a* and *SiATG9b*) showed highly expressed in darkness. The expression of the other *SiATG* genes was not changed significantly under this treatment. To monitor the response of *SiATG* genes in foxtail millet during nitrogen starvation, the expression of the 37 *SiATG* genes was analyzed in plants that had been subjected to nitrogen-free treatment for 1 h and for 24 h (Fig. 7b). After 1 h of treatment, the expression of most of the 37 *SiATG* genes was up-regulated, although the expression of *SiATG27* was down-regulated. After 24 h of starvation, the expression of many genes was elevated. Among 37 *SiATG* genes the expression level of *SiATG8a* was the highest after low nitrogen treatment 24 h, suggesting its potential role in tolerance to nutrient starvation.

### Overexpression of *SiATG8a* in rice conferred improved tolerance to nitrogen starvation

**Fig. 8:**
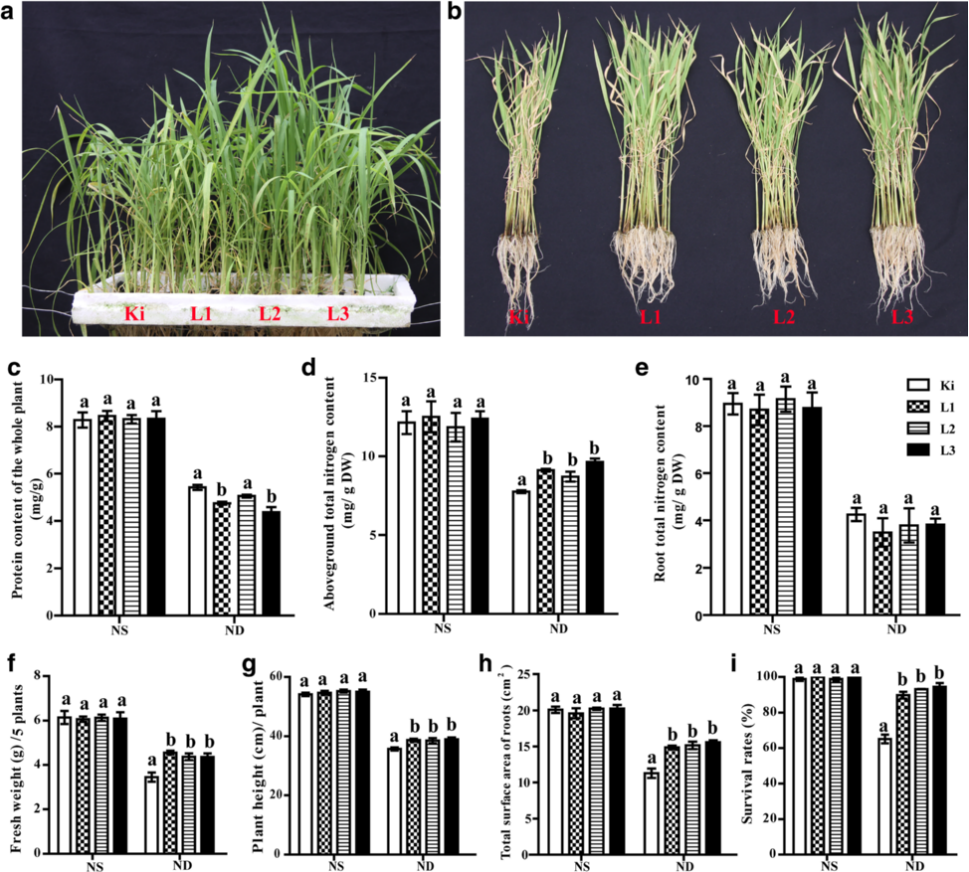
Heterologous expression of *SiATG8a* in rice enhanced tolerance to nitrogen starvation conditions. **a** and **b** 3-week old seedlings of control rice plants and transgenic plants grown in normal Hoagland solution were sub cultured in N-deficient liquid medium for 21 days. Statistical analysis of data for transgenic plants and wild type are shown for (**c**) protein content of the whole plant. **d** Aboveground total nitrogen content. **e** Root total nitrogen content. **f** Fresh weight. **g** Plant height. **h** Total surface area of roots. **i** Survival rate. For statistical analyses, the SAS 9.3 software package was used. Data presented in (**c-i**) are means ± SD from three independent experiments, and different letters above the columns indicate significant differences at the *P* ≤ 0.05 level. NS: N sufficient, ND: N deficient

We investigated the tolerance to low nitrogen stress in transgenic rice expressing *SiATG8a* (Fig. 8a, 8b). The heterologous expression of *SiATG8a* in three transgenic rice lines was confirmed. The transgenic rice plants showed markedly higher transcript levels (Additional file 1: Figure S2). Three T2 generation transgenic lines, referred to as L1, L2, and L3, were obtained and grown hydroponically under nitrogen-free conditions. After 21 days of nitrogen starvation, it was clear that the three transgenic lines had higher survival rates than the control rice plants (Fig. 8i). Although the total protein concentration did not differ between the transgenic plants and control rice under normal conditions, it was significantly decreased in the transgenic plants that were grown under nitrogen starvation conditions (Fig. 8c). Additionally, the nitrogen content in the aboveground parts of the plants and the fresh weight of the three transgenic lines were higher than in the control rice (Fig. 8d, f). We also found that the root total nitrogen content in the three transgenic rice lines was slightly reduced compared to the wild type, but the results of variance analysis showed that these differences were not statistically significant between transgenic plants and wild type under N-free conditions (Fig. 8e). Under nitrogen starvation treatment, the transgenic plants were, on average, taller than the control rice plants (Fig. 8g). qRT-PCR analysis of the 18 endogenous *OsATGs* showed that the expression of these genes did not differ between the wild type and transgenic plants (Additional file 1: Figure S3A, B). For the low nitrogen starvation treatment, 40 millet varieties were randomly chosen. As shown in Additional file 1: Figure S4, the expression levels of the *ATG8a* gene in 40 foxtail millet varieties were associated with plant height under nitrogen starvation. We also observed that nitrogen deficiency stress inhibited the growth of the foxtail millet above ground, but the range of the declines of the individual varieties was different. The foxtail millet variety #17 and 18 (Longgu 10 and Chihegu) had the highest values for these traits, but the expression of the *SiATG8a* gene was low (Additional file 1: Figure S4). The effect of gene expression and phenotype in the different millet varieties is also of relevance under nitrogen starvation.

## Discussion

### Heterologous expression of *SiATG8a* in rice enhances tolerance to nitrogen starvation

The *Arabidopsis* autophagy mutants, such as *atg4a4b-1*, *atg5-1, atg7-1, atg9-1*, *atg10-1* and *ATG18aRNAi* plants exhibited abnormal phenotypes compared to the wild-type plant (chlorosis, decreased bolting time, and early senescence) under nutrient starvation, which confirmed the important roles of autophagy in nutrient recycling [21, 22, 25–29]. Constitutive expression of *GmATG8c* in soybean callus cells enhanced nitrogen starvation tolerance and accelerated the growth of the calli. Transgenic *Arabidopsis* over-expressing *GmATG8c* increased the yield under the nitrogen starvation compared to wild type plants [37]. In our study, expression level of *SiATG8a* was the highest among 37 *SiATG* genes under nitrogen starvation condition, indicating its potential role in nutrient starvation responses. The *SiATG8a* transgenic plants appeared to be more robust than the wild type, indicating *SiATG8a* conferred transgenic plants tolerance to low nitrogen supply. N utilization is a complex process that consists of several steps, including uptake, translocation, assimilation, and remobilization [43]. Many results indicate that nutrient recycling could be precisely controlled by altering the timing and strength of autophagic processes, which would offer novel strategies for the improvement of N utilization in other crops [17, 24, 44]. Leaf soluble proteins can be rapidly degraded and are a major source of N in plants. Autophagy has recently been reported to be implicated in efficient nitrogen remobilization in senescent leaves, it facilitates protein degradation for nitrogen recycling in these organs [24, 45]. Tolerance to low nitrogen of *SiATG8a* might be related to the presumed enhanced protein degradation and utilization ability in senescent leaves. We found that the total nitrogen content of the aboveground parts in transgenic plants is significantly higher than that in control plants under low nitrogen condition (Fig. 8d). We also measured the total nitrogen content of roots and found that the total root nitrogen content in the three transgenic rice lines was slightly reduced compared to the wild type rice, but the difference was not statistically significant (Fig. 8e). There were no significant differences in the total nitrogen content for whole plants between transgenic and control plants (Additional file 1: Figure S5). These results indicated that the translocation of nitrogen from roots to aboveground organs might be enhanced in transgenic plants, which lead to strong tolerance to low nitrogen stress of transgenic plants. Further study will be necessary to characterize the molecular basis of how the *SiATG8a* promote influences nitrogen translocation from roots to aboveground organs in plants.

### The analysis of genes structure, phylogenetic classification, duplication and divergence rates of the *SiATG* genes

Many genes are known to be involved in autophagy. In yeast, 41 *ATGs* genes have been identified and confirmed to function in autophagy. In recent years, the autophagy-associated genes in multiple plant species have been identified. More than 30 *ATG* genes have been identified in *Arabidopsis thaliana*, *Zea mays*, *Oryza sativa* and *Nicotiana tabacum* genomes, demonstrating the evolutionarily conservation of the core ATG mechanism in different species [16–19]. A comprehensive search for autophagy- associated gene superfamily members from the PHYTOZOME database has revealed the presence of 37 *SiATG* genes, which is similar to the number of such genes in rice, tobacco and maize [16, 17, 19]. Unlike yeast where there is only a single copy, multiple copies of autophagy genes such as *ATG8*, exist in other species. For example, there are five, nine and eleven copies of *ATG8* in tobacco, *Arabidopsis*, and soybean, respectively [16, 19, 32]. The unrooted phylogenetic trees suggested that each SiATG protein sequence was highly similar to their homologues in other plant species, it clearly shows the derivation of statistically reliable pairs of possible orthologues proteins sharing similar functions from a common ancestor. These results are consistent with the present understanding of plant evolutionary history [41]. Close association of SiATG families with their counter-parts in other plants, expressions or functions for most of which have reported, may be an implication of sequence conservation and evidence to their similar biological roles. Such phylogeny-based function prediction has been applied for prediction of stress-responsive proteins in other species like rice, *Arabidopsis* and maize. Although SiATG proteins tended to cluster together in the tree with respect to their type and they were not equally distributed in the clades which may be due to the occurrence of duplication and divergence of SiATG genes.

In this study, the exon-intron structures of *ATG* genes identified in the foxtail millet indicated that the exon-intron structures were conserved in foxtail millet. The results of inter-genomic analyses between rice, sorghum, maize, and foxtail millet revealed that there is highly conserved colinearity, which supports a close evolutionary relationship among these grasses [39]. To further explore the origin and evolutionary process of *SiATG* genes, a comparative synteny map between foxtail millet and rice genomes was produced which showed that there are 22 genes unambiguously existing in both the foxtail millet and rice genomes. These results suggest that the majority of *SiATG* genes share a common ancestor with *OsATG* genes counterparts. Despite the close evolutionary relationship between the foxtail millet and rice, the chromosomes of grasses have undergone extensive rearrangement. There are some *SiATG* genes that were not mapped onto any colinear blocks with rice, such as *SiATG8a*. A possible explanation for this is that foxtail millet and rice chromosomes have undergone extensive rearrangements and fusions that led to selective gene loss, which makes the identification of chromosomal syntenies difficult [38]. It does not mean that these *ATG* genes from foxtail millet and rice do not share a common ancestor but may be linked to environmental conditions in which some millet genes might be more appropriate and result in a better performance than rice. Gene duplication events play a key role in the expansion of gene families [46]. Many genes are known to be involved in autophagy most of the foxtail millet duplications were generated in the whole genome duplication (WGD) event similar to that in Brachypodium, rice, sorghum and maize, this was estimated to have occurred approximately 70 MYA [39]. In this study, gene duplication analysis revealed that segmental and tandem duplicated events resulted in the generation of multiple copies of *SiATG* genes, suggesting that these events play a significant role in the expansion of the *SiATG* gene family. In addition, some duplicate genes are important in the evolution of new traits and in speciation [47]. In the long-term evolution process, some genes may have a relatively more important role. In genetics, the calculation of the Ka/Ks ratio is an important parameter when deciding whether Darwinian positive selection was involved in gene divergence [48]. In all organisms, the majority of non-synonymous substitutions are harmful mutation; only a minority neutral or favorable mutations. Darwinian positive selection will retain the benefit of the non-synonymous mutations, whereas, for the harmful non-synonymous mutations, purifying selection will remove them gradually. In the present study, the average ratio of Ka/Ks for segmentally duplicated gene pairs is 0.210, whereas the Ka/Ks value for tandemly duplicated gene pairs was 0.34, strongly indicating that the *SiATG* gene family underwent a strong purifying selection pressure as the Ka/Ks ratios of six duplicated pairs were <1. Comparative genomic analysis is a quick way to extrapolate genomic knowledge acquired in one taxon to a less-well-studied species [49]. Duplicate genes tend to diverge in coding and regulatory regions. Divergences in coding regions can change the function of the gene or may lead to the acquisition of new functions as a result of amino acid-altering substitutions and/or alterations in exon-intron structure, divergence in regulatory regions can result in change in expression pattern [50]. Previous research has revealed that the foxtail millet genome underwent whole-genome duplication similar to other grasses about 70 MYA [39]. The relatively higher rate of synonymous substitution between rice and foxtail millet *ATG* genes may point towards their earlier divergence around 36–95 MYA as compared to sorghum and maize *ATG* genes.

### Expression of *SiATG* genes in response to stress treatments

Under adverse environmental conditions, plants adopt various strategies to respond to particular stresses to enable growth and survival [51]. Many studies have shown that ATG proteins play key roles in plant responses to various environmental stresses [1, 20–22]. Cell autophagy generally remains at a low level in unstressed plants but can be induced when plants are subjected to adverse external stress. During such stress, plants require several phytohormone, including ABA, GA, MeJA and SA, which play key regulatory roles in different plant processes [52]. In our study, expression profile analysis demonstrated that all 37 *SiATGs* genes displayed variations in their expression behavior in response to one or more stresses, as shown in Additional file 1: Table S8. The complex expression patterns in response to one or more hormones implied that these 37 *SiATGs* may play different roles in response to multiple environmental stimuli. Oxidative, high salt and osmotic stress conditions can induce autophagy in plants. High salinity causes ion stress and drought usually leads to osmotic stress in the plant [53]. The accumulation of ROS (reactive oxygen species) and oxidized proteins in plant cells increased in plants grown under drought and salt stress conditions [54]. Autophagy is a macromolecular degradation pathway through which oxidized proteins can be voided. Transgenic RNAi-*AtATG18a* plants are more sensitive to ROS, salt and drought conditions than are wild type plants [25]. *AtATG8* and *OsATG10b* have been reported to play important roles in responses to salt and osmotic stresses [36, 55]. Recent studies have shown that autophagy plays an important role in nitrogen remobilization and seed production in *Arabidopsis* [44, 45]. During leaf senescence, nitrogen and fixed-carbon limitations, the levels of maize *ATG* transcripts and/ or the ATG8-lipid adduct were increased indicating that autophagy plays important role in nutrient remobilization [15]. In this study, most of the 37 *SiATGs* responded differentially to at least one stress (Fig. 6). *SiATG6a*, *SiATG8a*, *SiATG8c* and *SiATG9a* showed increased expression during carbohydrate starvation and *SiATG8a*, *SiATG8c* and *SiATG9a* were up-regulated under nitrogen starvation (Fig. 7a, b). Those results indicated that ATG genes are widely involved in various abiotic stress responses of plants in addition to low nitrogen stress in plants.

## Conclusion

A total of 37 putative *SiATG* genes were identified in foxtail millet through genome-wide analysis. We analyzed the chromosomal distribution, intron-exon structures, duplication and divergence rates, differential expression profiles under phytohormone, abiotic stresses, and nitrogen and carbon starvation treatments. Heterologous expression of *SiATG8a* in rice enhanced tolerance to nitrogen starvation in this study.

## Methods

### Plant materials and growth conditions

Seeds of foxtail millet were soaked in water and germinated at 28 °C for 2 days and then grown in Hoagland solution for 3 weeks. For the stress treatments, 3-week-old seedlings were exposed to high salt (100mM NaCl), drought (6 % PEG 6000), or hormones (5μM abscisic acid (ABA), 5μM salicylic acid (SA), 5μM methyl jasmonate (MeJA), and 5μM gibberellic acid (GA)) for 1 h and 24 h, respectively [56–58]. The carbon starvation and cold treatments were achieved, respectively, by keeping the seedlings in the dark for 48 h and keeping the seedlings at 4 °C for 1 h and 24 h. For the nitrogen-free treatment, foxtail millet seeds were surface sterilized and then germinated on MS medium (0.8 %w/v agar, 3 %sucrose) and cultured under a 16/8 h light/ dark photoperiod for 14 days. On day 15, seedlings were cultured on nitrogen-deficient liquid medium (modified MS medium with 5 mM KCl, 3 mM CaCl_2_, 1.5 mM MgSO_4_, 1.25mM KH_2_PO_4_, pH 5.8) for 1 h and 24 h. Control seedlings were grown in normal MS medium. For the nitrogen starvation treatment, 40 millet varieties were randomly chosen, the relative reduction values for the three replicates are presented. The list of the foxtail millet varieties examined in this study is shown in Additional file 1: Table S7. At the end of the treatments, all of the samples were frozen immediately in liquid nitrogen and stored at -80 °C prior to analysis.

### Bioinformatics analysis of the autophagy gene family in foxtail millet

Some *ATG*-like genes (*SiATGs*) were identified in the foxtail millet genomic database (PHYTOZOME v9.0 database, www.phytozome.net/) using ‘autophagy’ as a key word. Other foxtail millet ATGs, together with ATG proteins from yeast, rice, *Arabidopsis*, tobacco, and maize (sources detailed subsequently), were identified in the PHYTOZOME v9.0 database via BLASP using the ATG domain as a query. The coding sequences, genomic sequences, protein sequences and nucleotide sequences upstream of the initiation codon for transcription for the 37 Si*ATGs* were all downloaded from (www.phytozome.net/). Yeast ATG protein sequences were downloaded from Pfam26.0 (http://pfam.sanger.ac.uk/) [3–7]. The *Arabidopsis* ATG protein sequences were downloaded from the *Arabidopsis* information resource (https://www.arabidopsis.org/). The NtATG, ZmATG, and OsATG protein sequences were downloaded from (www.phytozome.net/) [16–19]. All redundant sequences were removed using the ‘decrease redundancy’ tool at http://web.expasy.org/decrease_redundancy/. Each putative *SiATG* gene sequence was checked by SMART (http://smart.embl-heidelberg.de/) to confirm the presence of the ATG domain. For nomenclature, the prefix `Si` for *Setaria italica* was used, followed by ‘*ATG’*. The serial number for each foxtail millet autophagy-associated genes member was assigned according to their relationship with the members of rice, *Arabidopsis*, tobacco, and maize autophagy-associated genes family. The subcellular localizations of the SiATG proteins were predicted using YLoc (http://abi.inf.uni-tuebingen.de/Services/YLoc/webloc.cgi), an interpretable web server for predicting the subcellular localization of proteins [59, 60].

### Phylogenetic analysis of *ATG* genes

To further investigate the evolutionary relationships of among the ATG proteins in various plants species, the phylogenetic trees of the *ATG* genes were constructed. Multiple sequence alignment of ATG protein sequences were conducted with the ClustalX 1.81 program using the default multiple alignment parameters. The unrooted phylogenetic tree were constructed using MEGA5.0 software with a maximum likelihood method using sequences from *S. italica* (Si), *N. tabacum* (Nt), *A. thaliana* (At), *O. sativa* (Os), and *Z. mays* (Zm) [16–19, 61]. Bootstrap analysis was performed with 1000 replicates to obtain a support value for each branch.

### Chromosomal location, gene structure analysis, and estimation of genomic distribution

All *SiATG* genes were mapped onto the nine foxtail millet chromosomes according to their ascending order of physical position (bp), from the short arm telomere to the long arm telomere, these were visualized using MapChart [62]. The exon-intron structures of the *SiATG* genes were determined by comparing the coding sequences (CDS) with their corresponding genomic sequences using the Gene Structure Display Server (GSDS) (http://gsds.cbi.pku.edu.cn/). Sequence duplications in foxtail millet, and the synteny between foxtail millet and rice, were established using the SynMap tool (https://genomevolution.org/CoGe/SynMap.pl) with the following settings: Blast Algorithm-Last, DAG Chainer options, and Merge syntenic Blocks, using the recommended parameters. Existing tandem duplications of the *ATG* genes in the foxtail millet genome were identified by manually evaluating the physical locations of genes within a region of 200 kb.

### Estimation of the synonymous and non-synonymous substitution rates of the *ATG* genes

Synonymous (Ks) and non-synonymous (Ka) substitution rates were estimated according to previously-described criteria [63]. Ks and Ka substitution rates were calculated using the CODEML program and confirmed with the GEvo tool (https://genomevolution.org/CoGe/SynMap.pl). The time (million years ago, MYA) of duplication and divergence time (T) was calculated using a synonymous mutation rate of λ substitutions per synonymous site per year as T = Ks/2λ (λ = 6.5 × 10^−9^) [64, 65].

### Total RNA isolation and quantitative real-time PCR

Total RNA from foxtail millet was isolated using Trizol reagent (Invitrogen, USA) according to the manufacturer’s instructions. The integrity of the RNA was confirmed by electrophoresis with 1.2 % agarose gels. The relative expression levels of the *SiATG* genes under various treatments were examined using quantitative real-time PCR (qRT-PCR). For each plant sample, 5μg of total RNA was reverse transcribed to cDNA in a 50μl reaction system using a PrimeScript 1st Strand cDNA Synthesis Kit (Transgen Biotech. Beijing, China). The primers for the qRT-PCR analysis were designed from a non-conserved region using Primer-BLAST (http://www.ncbi.nlm.nih.gov/tools/primer-blast/). Reverse primers were designed preferentially from the 3’-untranslated region wherever possible, as these regions are, in general, both relatively more unique than the coding sequence and closer to the reverse transcriptase (RT) start site. qRT-PCR was performed with three biological replicates, with three technical replicates per biological replicate. The primers used in these experiments are detailed in Additional file 1: Table S9.

### Construction of the *SiATG8a* gene vector and rice transformation

The *SiATG8a* coding region was amplified by PCR using the primers F1 (B*amH* I) and R1 (S*ac* I). The PCR product was then digested and ligated into the binary vector pMWB014 (driven by a ubiquitin promoter) to obtain the construct pMWB014-*SiATG8a*. This construct was transformed into *Oryza sativa* cv. Kitaake using Agrobacterium-mediated transformation [66]. Fifty transgenic rice lines were generated and confirmed by PCR using the F2 and R2 primers. Nine transgenic lines were analyzed for phenotypes. Similar phenotypes were observed for these nine lines, and data from three of these selected lines were chosen for presentation here. The transcription level of *SiATG8a* in the three selected transgenic rice lines was analyzed via qRT-PCR. The primers used in this analysis are detailed in Additional file 1: Table S10. The transcription levels of the endogenous *ATG* (i.e., *OsATG*) in the three selected transgenic rice lines and in wild-type rice were verified by qRT-PCR. The primers used in this analysis are detailed in Additional file 1: Table S11.

### Phenotypic analysis and measurement of nitrogen and protein content in transgenic rice plants

Seedlings of transgenic rice were grown in the greenhouse under a 16/8 photoperiod at 30 °C. For the nitrogen-free treatment experiment, 3-week-old seedlings of control rice and transgenic rice plants grown in normal Hoagland solution conditions were sub-cultured in nitrogen-deficient liquid medium (Hoagland hydroponic solution (pH 5.5), 5 mM KNO_3_, 5 mM Ca(NO_3_)_2_, 2 mM MgSO_4_, 1 mM NH_4_NO_3_). The nitrogen-free solution was prepared by replacing KNO_3_ and Ca(NO_3_)_2_ with KCl and CaCl_2_, respectively. After nitrogen starvation, plants that had green and healthy young leaves were considered to have survived, and the survival rate was calculated, using two green leaves as the standard for survival estimate. All experiments were repeated independently three times, and data were analyzed by Dunnett’s Test at the *P* ≤ 0.05 level. Total nitrogen content was determined by the Kjeldahl method [67]. Protein content was measured using the Bradford method [68].

## Additional file

Additional file 1: Figure S1. Expression analysis of autophagy-associated gene (*ATG*) in various foxtail millet organs. **Figure S2.** The transcription levels of *SiATG8a* in three transgenic rice lines relative to wild type rice. **Figure S3.** The transcription levels of 18 endogenous *ATG* in the three transgenic rice lines. **Figure S4.** *SiATG8a* expression and phenotype analysis in multiple foxtail millet varieties grown under nitrogen starvation conditions. **Figure S5.** The total nitrogen content for whole plants under normal and starvation conditions. **Table S1.** The autophagy-associated gene (*ATG*) homologue superfamily in foxtail millet. Information includes common names and locus names of the putative *SiATGs* in different versions of PHYTOZOME. **TableS2.** The autophagy-associated gene (*ATG*) homologue superfamily in foxtail millet. pI: isoelectric point, M: molecular weight. **Table S3.** Conserved motifs identified in the foxtail millet ATG family proteins using MEME software. **Table S4.** The Ka/Ks ratios and estimated divergence times for orthologous ATG proteins between foxtail millet and maize. **Table S5.** The Ka/Ks ratios and estimated divergence times for orthologous ATG proteins between foxtail millet and rice. **Table S6.** The Ka/Ks ratios and estimated divergence times for orthologous ATG proteins between foxtail millet and sorghum. **Table S7.** Foxtail millet varieties examined in this study. **Table S8.** Overview of the expression of *ATGs* in foxtail millet in response to treatment with various stresses. “+” and “-”indicate that the relative expression level of a given *ATGs* was up-regulated or down-regulated, respectively, in response to a given stress. The number represents the difference in expression expressed as a fold change. 0 means the absolute value are between 0 and 1;1 means ≥ 1 fold change; 2 means ≥ 2 fold change etc. **Table S9.** Primers used for the qRT-PCR analysis of the 37 *SiATG* genes. **Table S10.** A list of the primer sequences used for the cloning of *SiATG8a* and the PCR analysis of *SiATG8a* in transgenic rice. **Table S11.** Primers used for the for qRT-PCR analysis of endogenous *ATG* genes of rice. (DOCX 1530 kb)
