# Supplementary material for: Genome-wide analysis of autophagy-associated genes in foxtail millet (Setaria italica L.) and characterization of the function of SiATG8a in conferring tolerance to nitrogen starvation in rice
Source: BMC Genomics. 2016 Oct 12;17:797. doi: 10.1186/s12864-016-3113-4 (PMC5062844; doi:10.1186/s12864-016-3113-4)
Supplement: Additional file 1: Figure S1. — Expression analysis of autophagy-associated gene (ATG) in various foxtail millet organs. Figure S2. The transcription levels of SiATG8a in three transgenic rice lines relative to wild type rice. Figure S3. The transcription levels of 18 endogenous ATG in the three transgenic rice lines. Figure S4. SiATG8a expression and phenotype analysis in multiple foxtail millet varieties grown under nitrogen starvation conditions. Figure S5. The total nitrogen content for whole plants under normal and starvation conditions. Table S1. The autophagy-associated gene (ATG) homologue superfamily in foxtail millet. Information includes common names and locus names of the putative SiATGs in different versions of PHYTOZOME. TableS2. The autophagy-associated gene (ATG) homologue superfamily in foxtail millet. pI: isoelectric point, M: molecular weight. Table S3. Conserved motifs identified in the foxtail millet ATG family proteins using MEME software. Table S4. The Ka/Ks ratios and estimated divergence times for orthologous ATG proteins between foxtail millet and maize. Table S5. The Ka/Ks ratios and estimated divergence times for orthologous ATG proteins between foxtail millet and rice. Table S6. The Ka/Ks ratios and estimated divergence times for orthologous ATG proteins between foxtail millet and sorghum. Table S7. Foxtail millet varieties examined in this study. Table S8. Overview of the expression of ATGs in foxtail millet in response to treatment with various stresses. “+” and “-”indicate that the relative expression level of a given ATGs was up-regulated or down-regulated, respectively, in response to a given stress. The number represents the difference in expression expressed as a fold change. 0 means the absolute value are between 0 and 1;1 means ≥ 1 fold change; 2 means ≥ 2 fold change etc. Table S9. Primers used for the qRT-PCR analysis of the 37 SiATG genes. Table S10. A list of the primer sequences used for the cloning of SiATG8a and the PCR analysis of SiATG8a in transgenic [file 12864_2016_3113_MOESM1_ESM.docx]

**Additional Figure S1.** **The expression analysis of 37 foxtail millet autophagy-associated gene (*ATG*) in various organs.**Total RNA was extracted from different foxtail millet organs under normal growth conditions.The RT-PCR product generated with 37 SiATGs and actin gene specific primers. The bars represent the mean ± SD of the results from three separate experiments.Actin mRNA (AF288226.1) was used as an internal control.In the figure, root (A), stem (B) and leaf (C) are shown asyellow, blue and greenboxes.


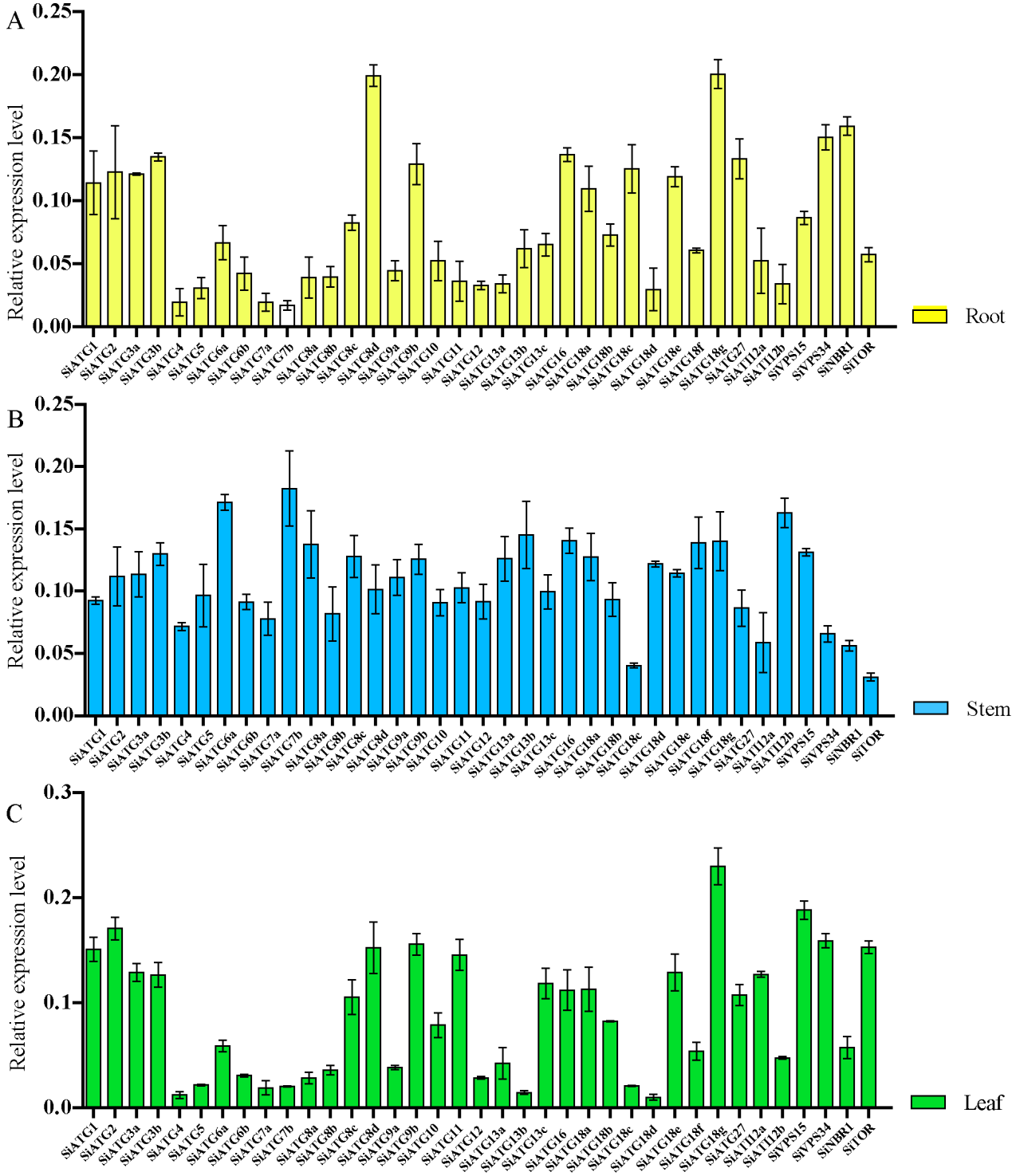


**Additional Figure S2.The transcription levels of SiATG8a in three transgenic rice lines relative to wild type rice.**Total RNA was extracted from rice seeding under normal growth conditions.The heterologous expression of *SiATG8a* in three transgenic rice lines were confirmed, the transgenic rice plants showed markedly higher transcript level. The bars represent the mean ± SD of the results from three separate experiments.

**
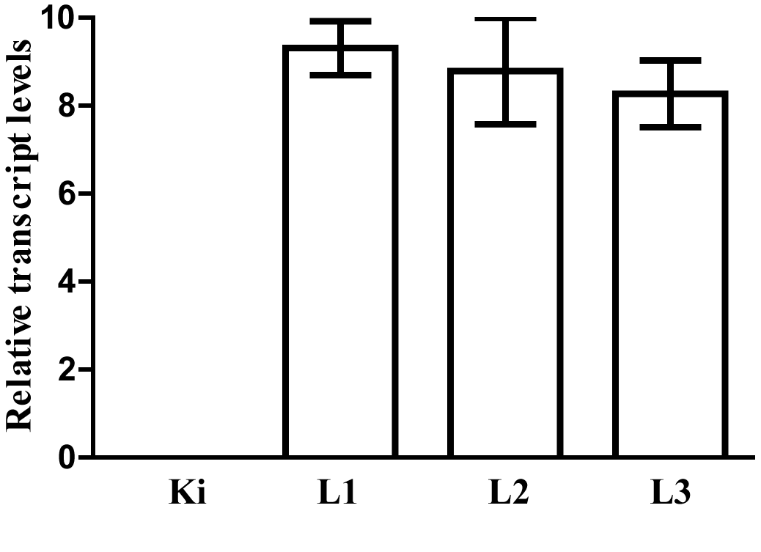
**

**Additional Figure S3.The transcription levels of 18 endogenous ATG in the three transgenic rice lines.** Total RNA was extracted from rice seeding under normal growth conditions (A) and nitrogen starvation condition (B). The bars represent the mean ± SD of the results from three biological replicates.NS: N sufficient, ND: N deficient.


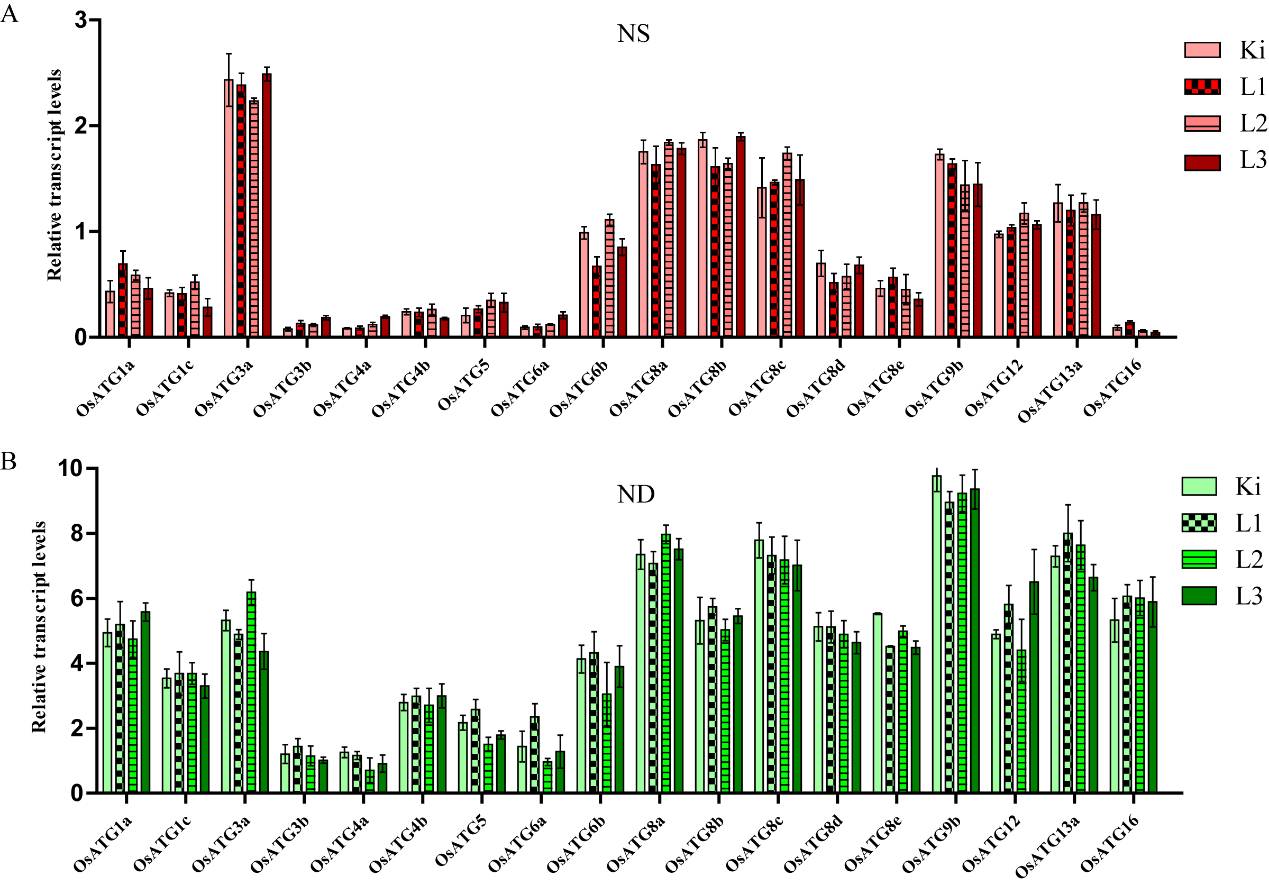


**Additional Figure S4. SiATG8a expression and phenotype analysis in multiple foxtail millet varieties grown under nitrogen starvation conditions.** During the growth stage, the plant-height of foxtail millet in normal growth conditions was higher than that in low nitrogen treatment. Comparing the plant height difference and the relationship between gene expressions. EL, expression level; PH, plant height difference; MV, millet variety.


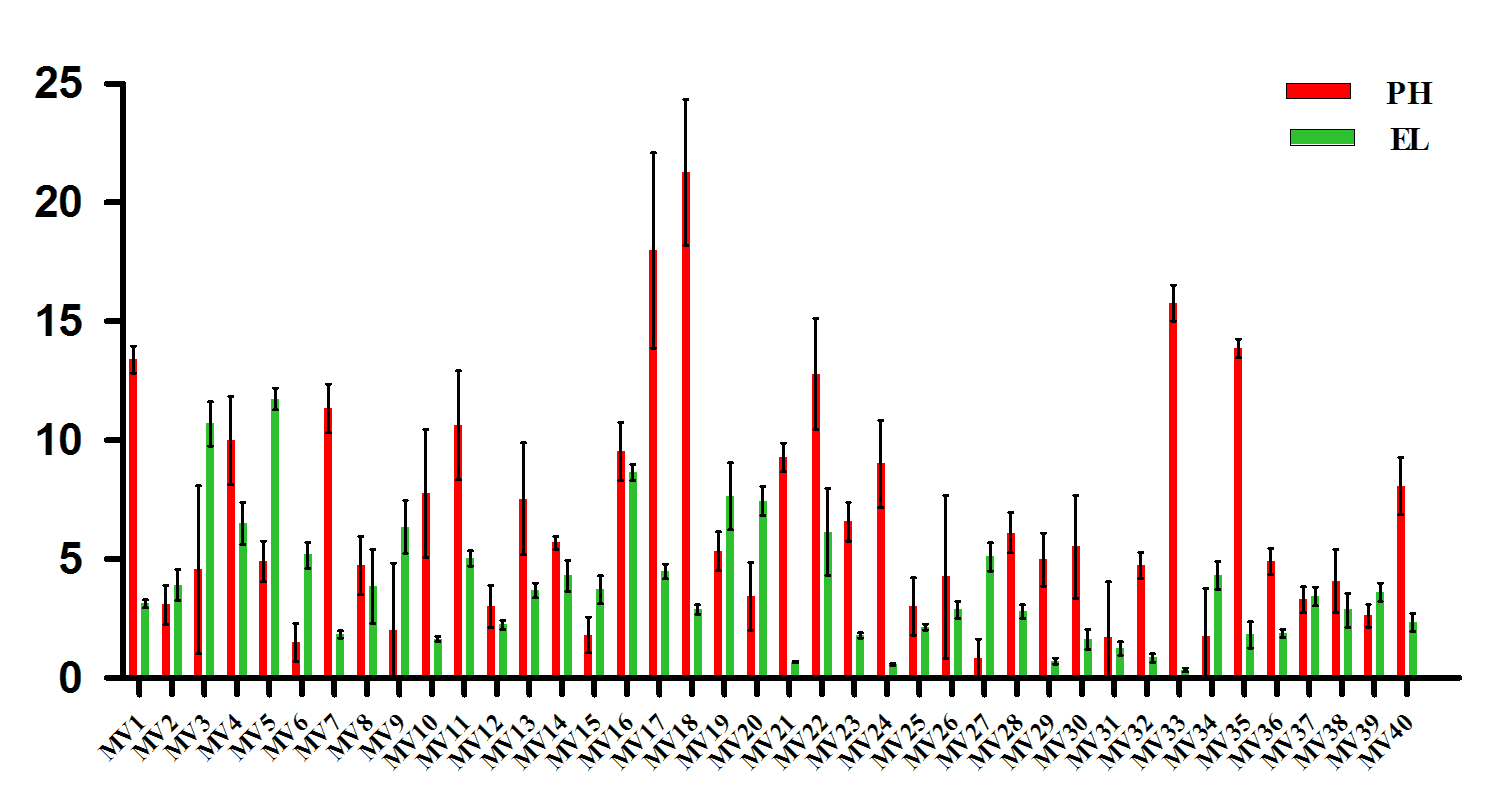


**Additional Figure S5.The total nitrogen content for whole plants under normal and starvation conditions.** Data presented are means ± SD from three independent experiments, and the same letter above the columns indicate no significant differences. NS: N sufficient, ND: N deficient.

**
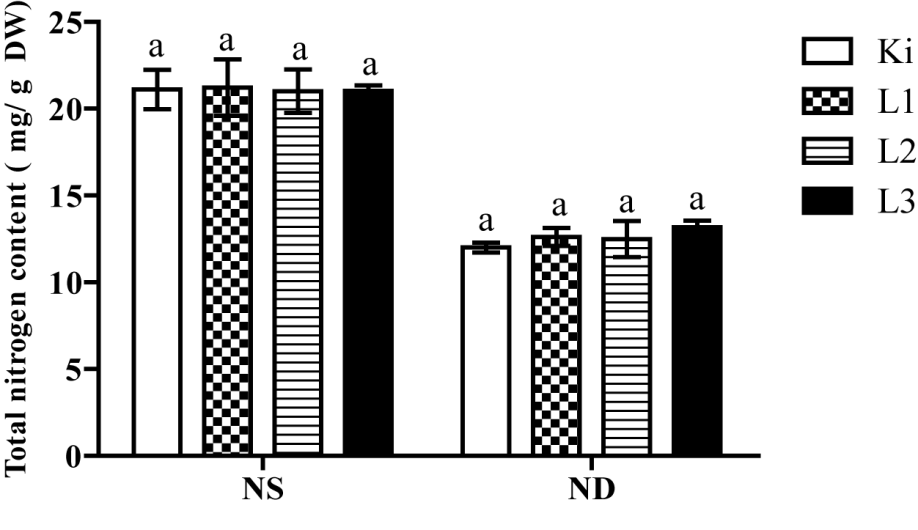
**

**Additional Table S1.The autophagy-associated gene (ATG) homologue superfamily in foxtail millet.**

| **Locus Name** | **Gene Name** | **Accession No.** | **ORF(aa)** | **Other Transcripts** | **Chr.** | **Location** |
| --- | --- | --- | --- | --- | --- | --- |
| Seita.9G459800 | SiATG1 | XP_004984919 | 702 | 1 | 9 | 50559416-50566408 |
| Seita.4G118900 | SiATG2 | XP_004965198 | 1929 | none | 4 | 11985390-11995977 |
| Seita.9G323500 | SiATG3a | XP_004983659 | 316 | none | 9 | 37272887-37277545 |
| Seita.9G562100 | SiATG3b | XP_012704218 | 603 | none | 9 | 57644951-57650398 |
| Seita.3G012000 | SiATG4 | XP_004960034 | 480 | none | 3 | 641351-645195 |
| Seita.1G112300 | SiATG5 | XP_004952117 | 363 | 1 | 1 | 9838477-9842262 |
| Seita.5G434000 | SiATG6a | XP_004971111 | 500 | none | 5 | 45184795-45189199 |
| Seita.5G272100 | SiATG6b | XP_012701217 | 483 | none | 5 | 33349085-33355731 |
| Seita.5G225600 | SiATG7a | XP_004969164 | 970 | none | 5 | 28556461-28563079 |
| Seita.5G225500 | SiATG7b | XP_004969162 | 1090 | none | 5 | 28528780-28543103 |
| Seita.6G036300 | SiATG8a | KX539300 | 119 | none | 6 | 2737490-2740232 |
| Seita.7G243500 | SiATG8b | XP_004976866 | 121 | 1 | 7 | 30401534-30404726 |
| Seita.4G053000 | SiATG8c | XP_004964683 | 118 | none | 4 | 3955705-3957649 |
| Seita.2G329800 | SiATG8d | XP_004957839 | 119 | 1 | 2 | 41654965-41657049 |
| Seita.9G189200 | SiATG9a | XP_004982580 | 933 | none | 9 | 13210654-13216962 |
| Seita.9G469500 | SiATG9b | XP_004985007 | 881 | none | 9 | 51220554-51227870 |
| Seita.7G153000 | SiATG10 | XP_004976100 | 216 | 3 | 7 | 23902365-23904362 |
| Seita.1G063400 | SiATG11 | XP_012703924 | 1132 | none | 1 | 5993763-5999108 |
| Seita.1G338600 | SiATG12 | XP_002454650 | 91 | none | 1 | 39548934-39551215 |
| Seita.8G043000 | SiATG13a | XP_004978757 | 586 | none | 8 | 3350856-3356266 |
| Seita.7G182900 | SiATG13b | XP_004976381 | 516 | none | 7 | 26035922-26040392 |
| Seita.1G252500 | SiATG13c | XP_004953275 | 540 | none | 1 | 32769713-32774989 |
| Seita.9G095000 | SiATG16 | XP_004981800 | 507 | 1 | 9 | 5702454-5705238 |
| Seita.9G171500 | SiATG18a | XP_012698175 | 1644 | none | 9 | 11654563-11665642 |
| Seita.3G100300 | SiATG18b | XP_004963506 | 382 | none | 3 | 6562814-6565047 |
| Seita.1G348000 | SiATG18c | XP_004954188 | 373 | none | 1 | 40224648-40228162 |
| Seita.5G442100 | SiATG18d | XP_004971179 | 449 | none | 5 | 45691509-45695400 |
| Seita.5G118500 | SiATG18e | XP_012701805 | 417 | none | 5 | 9929985-9935043 |
| Seita.3G247300 | SiATG18f | XP_004962126 | 1003 | none | 3 | 21223477-21230699 |
| Seita.5G343900 | SiATG18g | XP_004970215 | 868 | none | 5 | 38890009-38896087 |
| Seita.9G437800 | SiATG27 | XP_012704303 | 289 | none | 9 | 49019405-49025330 |
| Seita.5G205400 | SiVTI12a | XP_004969011 | 221 | none | 5 | 26395673-26399110 |
| Seita.5G289300 | SiVTI12b | XP_004969702 | 221 | none | 5 | 34751401-34755994 |
| Seita.1G351600 | SiVPS15 | XP_004954223 | 1471 | 9 | 1 | 40471966-40487821 |
| Seita.6G091400 | SiVPS34 | XP_004973055 | 803 | 1 | 6 | 8430199-8438415 |
| Seita.1G216500 | SiNBR1 | XP_004952961 | 853 | 1 | 1 | 29580088-29585110 |
| Seita.3G273900 | SiTOR | XP_004962343 | 2474 | 1 | 3 | 24814116-24848642 |

**Additional Table S2. The informations of the autophagy-associated gene (*ATG*) homologue superfamily in foxtail millet**

| **Locus name** | **pI** | **M** | **Predicted location** |
| --- | --- | --- | --- |
| SiATG1 | 6.42 | 77254.30 | cytoplasm |
| SiATG2 | 5.31 | 212286.5 | cytoplasm |
| SiATG3a | 4.53 | 35662.86 | cytoplasm |
| SiATG3b | 6.02 | 67974.42 | cytoplasm |
| SiATG4 | 4.93 | 52291.65 | Nucleus |
| SiATG5 | 5.03 | 40327.61 | Plasma membrane |
| SiATG6a | 6.35 | 56171.54 | cytoplasm |
| SiATG6b | 5.57 | 54614.94 | Mitochondrion |
| SiATG7a | 5.53 | 106196.72 | cytoplasm |
| SiATG7b | 5.32 | 55631.33 | Cytoplasm |
| SiATG8a | 9.12 | 13730.90 | cytoplasm |
| SiATG8b | 7.88 | 13972.15 | cytoplasm |
| SiATG8c | 8.49 | 11592.63 | Extracellular space |
| SiATG8d | 8.75 | 13564.72 | cytoplasm |
| SiATG9a | 6.35 | 107007.69 | Plasma membrane |
| SiATG9b | 6.23 | 100913.55 | Plasma membrane |
| SiATG10 | 4.79 | 24161.95 | cytoplasm |
| SiATG11 | 5.66 | 126956.55 | cytoplasm |
| SiATG12 | 8.03 | 10267.83 | cytoplasm |
| SiATG13a | 6.75 | 64738.41 | Nucleus |
| SiATG13b | 6.98 | 55998.92 | Nucleus |
| SiATG13c | 9.03 | 57632.33 | Nucleus |
| SiATG16 | 6.37 | 55594.8 | Nucleus |
| SiATG18a | 4.79 | 24161.95 | cytoplasm |
| SiATG18b | 8.70 | 41692.27 | cytoplasm |
| SiATG18c | 8.60 | 40604.33 | cytoplasm |
| SiATG18d | 5.36 | 48499.49 | Nucleus |
| SiATG18e | 8.28 | 46064.41 | cytoplasm |
| SiATG18f | 5.89 | 107776.22 | Mitochondrion |
| SiATG18g | 6.18 | 92126.55 | cytoplasm |
| SiATG27 | 7.49 | 29401.98 | Plasma membrane |
| SiVTI12a | 9.51 | 24667.3 | Cytoplasm |
| SiVTI12b | 9.08 | 24856.6 | Cytoplasm |
| SiVPS15 | 7.64 | 163787.4 | Cytoplasm |
| SiVPS34 | 5.96 | 91955.6 | Cytoplasm |
| SiNBR1 | 5.91 | 92570.3 | Cytoplasm |
| SiTOR | 6.64 | 278114.1 | Nucleus |

**Additional Table S3.Conserved motifs identified in the foxtail millet ATG family proteins using MEME software.**

| **Motif No.** | **Sites** | **E-value** | **Amino acid sequence composition of motif** | **Width**  **(aa)** |
| --- | --- | --- | --- | --- |
| Motif1 | 4 | 2.6e-069 | R[IL][KH]LS[AP][EG][KT]A[IL]F[IV]FV[KN]NTLP[PQ]TA[AS]LM[SG][AS][IV]Y[ED][EA][NY]KD[EAK]DGFLYM[TC]YS[GS]E[NK]TFG | 50 |
| Motif2 | 6 | 7.3e-051 | [AL][GQ][GT]LG[CA][ED][ILV][AL][KR][IN]L[AMV][LD][SA]G[VF][KR][KS][LV][TH][VL][HIV]D[DS][GD][KRT][VI][DV][VL][SW][ND]L[NS][RS][QN][FS][LF][FLY][RST][CE][DK]D[VR]G[QA][NPS][KR]A[QT] | 50 |
| Motif3 | 4 | 1.8e-040 | [YV][PF][DG][RY][IL][PQ]VIVE[KR][AF][EGS][RK][ST][DN][ILV]P[DEQ][IM][DE]K[KR]KYLVP[AC]D[LM][TP]VGQF[IV][FY][VI][VL]R | 41 |
| Motif4 | 5 | 1.2e-036 | [GHY][TVN][HCLR]L[QY][EK][VL][RQY]RG[AILMR][DT][KNQRS]A[EVD]I[YKQ][SD][IL][AS][FL]S[PDHN][NDF][SVL][QE][WY][LI][AMS][VI][SCV]S[DS][KR]GT[CILSV]H[IVL]F | 40 |
| Motif5 | 6 | 6.7e-028 | AHTSPI[AS][AC][ML][AC][FL][DST]P[DS]GTLL[AV]TAS[VT][KH]GT[LN][IV][RN] | 29 |
| Motif6 | 2 | 5.7e-027 | KCNTQMVIPHLTENYGASRDPPEKQAPMCTVHSFPHNIDHCLTWARSEFE | 50 |
| Motif7 | 2 | 3.6e-025 | PDLDIFFERLYEYFCAKGLRCIITKWIIEILNV[LT]FMVC[AC]IGFFFLFVDWD | 50 |
| Motif8 | 4 | 5.4e-026 | [VH][KEV][IV]E[TW]DE[IK][AN][AT][AS][LW][GS][QS]A[AS][LV][DL][DL][AH][AT][MV][AI][EQ][EY][LFY][IT][AP]K[FL][EQ][SY][IR][INS]K[IT][HL][PA][MPS]G[FS][HY][MP][NR][PIV][IT][DQ][FI][EHN][KN] | 50 |
| Motif9 | 4 | 1.6e-024 | [KL][EP]K[GAM]L[EN][AW][TY][LS][IN][SW]C[GI][FT][QS][LS][LM][FY][DN]S[KM]F[CP][RV][HGR]K[ED][FR]L[DT][KS][KP][VDS]V[DL][KV][AK]R[EL][IV] | 40 |
| Motif10 | 2 | 2.7e-024 | ECLDRDKCETFQDCITWARLKFEDYFANRVKQLTFTFPEDAMTSSGAPFW | 50 |
| Motif11 | 6 | 3.4e-023 | P[NRT][VK]V[MR][FI][WY][DS][DL][RH][QT][HS]R[CY][IV][GH]ELRFRSAV[RY][AS]VR[LC]S[PR][DR][RY][ILV][AV]VVL[AE]R[KQ]I[FY][VC][YF][DN][AFL][AT][DT] | 49 |
| Motif12 | 2 | 2.2e-022 | KVEDYRNTFANLAIPLFS[IM]AEPVPPKTIKHQDMSWTVWDRWTITGNITLR | 50 |
| Motif13 | 3 | 2.6e-021 | LSF[MI][KR]G[IV]LP[KD]YF[SH]SEWS[FV]AQF[RH]L[PH]E[GTV][TE][RQ]Y[IV][VA]AFG[AEH][QE][NK][TN][VT][MV] | 40 |
| Motif14 | 2 | 1.2e-020 | YNHPS[AT]ASSRRWSNLSRWI[FL]REYNEV[DE]HFFRHRMNN[CS][AT][LV]HSLNYLKQFPT | 50 |
| Motif15 | 2 | 1.5e-019 | CYFCNDVVAPVDSVSNRTLDQQCTVTRPGLACIASGRAADLFTRMLHHPD | 50 |
| Motif16 | 2 | 3.0e-019 | QFLFRDWNIGQPKSTVAATAAM[AT]INPKLHVEALQNRASPETENVFNDAFW | 50 |
| Motif17 | 2 | 3.5e-019 | IDKKLLQHFASGSRAVLNPMAAMFGGIVGQEVVKACSGKFHPLYQFFYFD | 50 |
| Motif18 | 2 | 5.2e-019 | NFQAVVFTDISIEKAVEFDDYCHSHQPPIAFIKSEVRGLFGSVFCDFGPE | 50 |
| Motif19 | 2 | 5.6e-019 | GNCTACSIAVLSEYRRRGLDFVMQAINYPTYLKDL[AT]GISNLKKPD[PT]CPKM | 50 |
| Motif20 | 2 | 3.0e-017 | [HQ][HQ]TH[HY]MPKRWRGKESSELVR[KR]E[FY]ETLFQYTI[IT]MLLEEMASIFITPYL[FL]IF | 50 |

**Additional Table S4.The Ka/Ks ratios and estimated divergence time for orthologous ATG proteins between foxtail millet and maize.**

| **Foxtail millet--Maize** | | | | **Ks** | **Ka** | **Ka/Ks** | **Date(million years)** |
| --- | --- | --- | --- | --- | --- | --- | --- |
| **ID(Name)** | **Chr.** | **ID(Name)** | **Chr.** |  |  |  |  |
| SiATG8b | 7 | GRMZM2G419694_T01 | 10 | 0.2928 | 0.0106 | 0.0362 | 22.52 |
| SiATG8b | 7 | GRMZM2G076826_T01 | 2 | 0.2757 | 0.011 | 0.0399 | 21.21 |
| SiATG8d | 2 | GRMZM2G336871_T02 | 2 | 0.5904 | 0.0229 | 0.0388 | 45.42 |
| SiATG9b | 9 | GRMZM2G035461_T01 | 1 | 0.2768 | 0.0512 | 0.1850 | 21.29 |
| SiATG27 | 9 | GRMZM2G110000_T02 | 1 | 0.2982 | 0.0965 | 0.3236 | 22.94 |
| SiATG6a | 5 | GRMZM2G027857_T01 | 8 | 0.2675 | 0.0677 | 0.2531 | 20.58 |
| SiATG6a | 5 | GRMZM2G092112_T01 | 3 | 0.2584 | 0.0293 | 0.1134 | 19.88 |
| SiATG13b | 7 | GRMZM2G000973_T01 | 10 | 0.3649 | 0.0974 | 0.2669 | 28.07 |
| SiATG13b | 7 | GRMZM5G825909_T01 | 2 | 0.3048 | 0.0785 | 0.2575 | 23.45 |
| SiATG13b | 7 | GRMZM2G000973_T01 | 10 | 0.3649 | 0.0974 | 0.2669 | 28.07 |
| SiATG13c | 1 | GRMZM2G000973_T01 | 10 | 1.0705 | 0.2629 | 0.2456 | 82.35 |
| SiATG13c | 1 | GRMZM2G129675_T01 | 4 | 0.3425 | 0.0459 | 0.1340 | 26.35 |
| SiATG13c | 1 | GRMZM2G109348_T01 | 5 | 0.3218 | 0.0617 | 0.1917 | 24.75 |
| SiATG16 | 9 | GRMZM2G078252_T02 | 1 | 0.3575 | 0.0343 | 0.0959 | 27.50 |
| SiATG11 | 1 | GRMZM2G143445_T01 | 4 | 0.3107 | 0.0831 | 0.2675 | 23.90 |
| SiATG3a | 9 | GRMZM5G818887_T01 | 9 | 0.2981 | 0.0331 | 0.1110 | 22.93 |
| SiATG5 | 1 | GRMZM2G098420_T02 | 5 | 0.2996 | 0.0762 | 0.2543 | 23.05 |
| SiATG10 | 7 | GRMZM2G066059_T07 | 10 | 0.3732 | 0.1415 | 0.3792 | 28.71 |
| SiATG18f | 3 | GRMZM2G078468_T02 | 8 | 0.2259 | 0.0427 | 0.1890 | 17.38 |
| SiATG18g | 5 | GRMZM2G116700_T01 | 3 | 0.2809 | 0.0565 | 0.2011 | 21.61 |
| SiATG18c | 1 | GRMZM2G018573_T01 | 5 | 0.2414 | 0.0525 | 0.2175 | 18.57 |
| SiATG18d | 5 | GRMZM2G122607_T01 | 8 | 0.3762 | 0.0515 | 0.1369 | 28.94 |
| SiATG18e | 5 | GRMZM2G069177_T01 | 3 | 0.3602 | 0.0324 | 0.0900 | 27.71 |
| SiATG18e | 5 | GRMZM2G143211_T01 | 8 | 0.3195 | 0.0472 | 0.1477 | 24.58 |
| SiATG12 | 1 | GRMZM5G842517_T02 | 5 | 0.3891 | 0.005 | 0.0129 | 29.93 |
| SiATG3b | 9 | GRMZM2G105415_T01 | 1 | 0.3733 | 0.1133 | 0.3035 | 28.72 |
| SiVTI12a | 5 | GRMZM2G010836_T02 | 8 | 0.2825 | 0.0562 | 0.1989 | 21.73 |
| SiVTI12b | 5 | GRMZM2G111611_T01 | 3 | 0.3455 | 0.01 | 0.0289 | 26.58 |
| SiVPS15 | 1 | GRMZM2G179662_T01 | 5 | 0.2691 | 0.0633 | 0.2352 | 20.70 |
| SiNBR1 | 1 | GRMZM2G092447_T01 | 5 | 0.3969 | 0.1078 | 0.2716 | 30.53 |
| SiTOR | 3 | GRMZM2G049342_T01 | 6 | 0.3597 | 0.0486 | 0.1351 | 27.67 |
| Average | | | | 0.3512 | 0.0641 | 0.1826 | 27.02 |

**Additional Table S5. The Ka/Ks ratios and estimated divergence time for orthologous ATG proteins between foxtail millet and rice.**

| **Foxtail millet--Rice** | | | | **Ks** | **Ka** | **Ka/Ks** | **Date(million years)** |
| --- | --- | --- | --- | --- | --- | --- | --- |
| **ID(Name)** | **Chr.** | **ID(Name)** | **Chr.** |  |  |  |  |
| SiATG8b | 7 | OsATG8b | Chr4 | 0.5879 | 0.0329 | 0.0560 | 45.22 |
| SiATG8b | 7 | OsATG8c | Chr8 | 1.1672 | 0.0223 | 0.0191 | 89.78 |
| SiATG8d | 2 | OsATG8a | Chr7 | 0.6924 | 0.0496 | 0.0716 | 53.26 |
| SiATG9b | 9 | OsATG9a | Chr3 | 0.6299 | 0.1072 | 0.1702 | 48.45 |
| SiATG13b | 7 | OsATG13b | Chr4 | 0.64 | 0.1389 | 0.2170 | 49.23 |
| SiATG13c | 1 | OsATG13a | Chr2 | 0.6321 | 0.0934 | 0.1478 | 48.62 |
| SiATG3a | 9 | OsATG3b | Chr10 | 0.6731 | 0.094 | 0.1397 | 51.78 |
| SiATG5 | 1 | OsATG5 | Chr2 | 0.476 | 0.1268 | 0.2664 | 36.62 |
| SiATG7a | 5 | OsATG7 | Chr1 | 0.5469 | 0.1344 | 0.2457 | 42.07 |
| SiATG10 | 7 | OsATG10a | Chr4 | 0.5841 | 0.1583 | 0.2710 | 44.93 |
| SiATG18f | 3 | OsATG18f | Chr5 | 0.5164 | 0.0825 | 0.1598 | 39.72 |
| SiATG18g | 5 | OsATG18e | Chr1 | 0.533 | 0.1226 | 0.2300 | 41.00 |
| SiATG18c | 1 | OsATG18a | Chr2 | 0.4899 | 0.1537 | 0.3137 | 37.68 |
| SiATG18d | 5 | OsATG18c | Chr1 | 0.6986 | 0.046 | 0.0658 | 53.74 |
| SiATG18e | 5 | OsATG18b | Chr1 | 0.6146 | 0.0559 | 0.0910 | 47.28 |
| SiATG18b | 3 | OsATG18d | Chr5 | 1.2368 | 0.2866 | 0.2317 | 95.14 |
| SiATG12 | 1 | OsATG12 | Chr6 | 0.869 | 0.1331 | 0.1532 | 66.85 |
| SiATG6b | 5 | OsATG6a | Chr1 | 0.5139 | 0.1817 | 0.3536 | 39.53 |
| SiATG3a | 9 | OsATG3b | Chr10 | 0.6731 | 0.094 | 0.1397 | 51.78 |
| SiATG1 | 9 | OsATG1a | Chr3 | 0.4893 | 0.1417 | 0.2896 | 37.64 |
| SiATG13c | 1 | OsATG13b | Chr4 | 0.9507 | 0.2123 | 0.2233 | 73.13 |
| SiATG13b | 7 | OsATG13a | Chr2 | 0.9408 | 0.2024 | 0.2151 | 72.37 |
| SiATG18b | 3 | OsATG18b | Chr1 | 1.7961 | 0.2913 | 0.1622 | 138.16 |
| SiATG18e | 5 | OsATG18d | Chr5 | 1.1793 | 0.2644 | 0.2242 | 90.72 |
| SiATG4 | 3 | OsATG4b | Chr4 | 0.657 | 0.0958 | 0.1458 | 50.54 |
| SiVTI12b | 5 | LOC_Os01g51120 | Chr1 | 0.3668 | 0.029 | 0.0791 | 28.22 |
| SiVPS15 | 1 | LOC_Os02g55340 | Chr2 | 0.6031 | 0.1213 | 0.2011 | 46.39 |
| SiVPS34 | 6 | LOC_Os02g38050 | Chr2 | 0.8225 | 0.2077 | 0.2525 | 63.27 |
| Average | | | | 0.7350 | 0.1314 | 0.1834 | 56.54 |

**Additional Table S6. The Ka/Ks ratios and estimated divergence time for orthologous ATG proteins between foxtail millet and sorghum.**

| **Foxtail millet--Sorghum** | | | | **Ks** | **Ka** | **Ka/Ks** | **Date(million years)** |
| --- | --- | --- | --- | --- | --- | --- | --- |
| **ID(Name)** | **Chr.** | **ID(Name)** | **Chr.** |  |  |  |  |
| SiATG8b | 7 | Sb07g005440 | 7 | 0.8899 | 0.0219 | 0.0246 | 68.45 |
| SiATG8d | 2 | Sb02g034500 | 2 | 0.4997 | 0.0254 | 0.0508 | 38.44 |
| SiATG9a | 9 | Sb01g016890 | 1 | 0.2364 | 0.0636 | 0.2690 | 18.18 |
| SiATG9b | 9 | Sb01g041090 | 1 | 0.309 | 0.0444 | 0.1437 | 23.77 |
| SiATG27 | 9 | Sb01g038200 | 1 | 0.4647 | 0.1133 | 0.2438 | 35.75 |
| SiATG6a | 5 | Sb03g044340 | 3 | 0.238 | 0.0276 | 0.1160 | 18.31 |
| SiATG6b | 5 | Sb03g031280 | 3 | 0.3518 | 0.1915 | 0.5443 | 27.06 |
| SiATG13a | 8 | Sb05g003870 | 5 | 0.3411 | 0.0887 | 0.2600 | 26.24 |
| SiATG13b | 7 | Sb04g033470 | 4 | 1.0171 | 0.2001 | 0.1967 | 78.24 |
| SiATG13c | 1 | Sb06g023870 | 6 | 0.9665 | 0.2282 | 0.2361 | 74.35 |
| SiATG11 | 1 | Sb04g005420 | 4 | 0.3058 | 0.072 | 0.2354 | 23.52 |
| SiATG5 | 1 | Sb04g001330 | 4 | 0.2683 | 0.0655 | 0.2441 | 20.64 |
| SiATG7a | 5 | Sb03g027840 | 3 | 0.2189 | 0.0741 | 0.3385 | 16.84 |
| SiATG10 | 7 | Sb06g021450 | 6 | 0.3926 | 0.1661 | 0.4231 | 30.20 |
| SiATG18f | 3 | Sb09g019970 | 9 | 0.2285 | 0.0347 | 0.1519 | 17.58 |
| SiATG18g | 5 | Sb03g036750 | 3 | 0.2323 | 0.0397 | 0.1709 | 17.87 |
| SiATG18c | 1 | Sb04g035650 | 4 | 0.4087 | 0.1579 | 0.3863 | 31.44 |
| SiATG18d | 5 | Sb03g044990 | 3 | 0.3884 | 0.0359 | 0.0924 | 29.88 |
| SiATG18e | 5 | Sb03g004650 | 3 | 0.2937 | 0.0261 | 0.0889 | 22.59 |
| SiATG18b | 3 | Sb09g005066 | 9 | 0.4435 | 0.1524 | 0.3436 | 34.12 |
| SiATG12 | 1 | Sb04g034790 | 4 | 0.2351 | 0.0002 | 0.0009 | 18.08 |
| SiATG3b | 9 | Sb01g049100 | 1 | 0.304 | 0.1004 | 0.3303 | 23.38 |
| SiATG2 | 4 | Sb10g009700 | 10 | 0.2771 | 0.0805 | 0.2905 | 21.32 |
| SiATG18a | 9 | Sb01g014880 | 1 | 0.2299 | 0.0179 | 0.0779 | 17.68 |
| SiVTI12a | 5 | Sb03g190600 | 3 | 0.2118 | 0.0481 | 0.2271 | 16.29 |
| SiVTI12b | 5 | Sb03g273100 | 3 | 0.2994 | 0.0082 | 0.0274 | 23.03 |
| SiVPS15 | 1 | Sb04g331100 | 4 | 0.2427 | 0.0519 | 0.2138 | 18.67 |
| SiNBR1 | 1 | Sb04g200200 | 4 | 0.3811 | 0.0952 | 0.2498 | 29.32 |
| SiTOR | 3 | Sb09g109200 | 9 | 0.2307 | 0.0165 | 0.0715 | 17.75 |
| Average | | | | 0.3760 | 0.0775 | 0.2061 | 28.93 |

**Additional Table S7. Foxtail millet varieties examined in this study.**

| MV1 | Jinsuigu 1 | MV21 | Zijieqi |
| --- | --- | --- | --- |
| MV2 | Huangdanzigu | MV22 | Jiyechong 1 |
| MV3 | Jigu 12 | MV23 | Longgu 8 |
| MV4 | Longgu 3 | MV24 | Longgu4 |
| MV5 | Longgu6 | MV25 | Chigu9 |
| MV6 | Shuxiang 1 | MV26 | Chigu8 |
| MV7 | Daobaqi | MV27 | Shanxihonggu |
| MV8 | Jigu21 | MV28 | Jigu 17 |
| MV9 | Bachagu | MV29 | Chigu6 |
| MV10 | Jigu18 | MV30 | Chigu10 |
| MV11 | Jigu20 | MV31 | Chigu 4 |
| MV12 | K325 | MV32 | Caopiyidaobaqi |
| MV13 | Huangjinmiao | MV33 | Honggaigu |
| MV14 | Longli 2 | MV34 | Huagu |
| MV15 | Longgu 9 | MV35 | Huangshatuchugu |
| MV16 | Pin 141 | MV36 | Lugu 9 |
| MV17 | Longgu 10 | MV37 | Jigu 24 |
| MV18 | Chihegu | MV38 | Datonggu |
| MV19 | Jigu15 | MV39 | Hongzhangu |
| MV20 | Dacaopi | MV40 | Longgu5 |

**Additional Table S8**. **Overview of the expression of ATGs in foxtail millet in response to treatment with various stresses.**

| Gene Name | Starvation | | | Hormone | | | | | | | | Abiotic | | | | | |
| --- | --- | --- | --- | --- | --- | --- | --- | --- | --- | --- | --- | --- | --- | --- | --- | --- | --- |
|  | Darkness | ND | | GA | | MeJA | | SA | | ABA | | Salt | | Drought | | Cold | |
|  | 48h | 1h | 24h | 1h | 24h | 1h | 24h | 1h | 24h | 1h | 24h | 1h | 24h | 1h | 24h | 1h | 24h |
| SiATG1 | +1 | 0 | 0 | 0 | 0 | 0 | +1 | 0 | 0 | +1 | +2 | 0 | +2 | -1 | +1 | 0 | 0 |
| SiATG2 | +1 | 0 | +1 | 0 | 0 | -1 | +1 | 0 | 0 | 0 | +2 | 0 | +2 | -2 | +1 | 0 | 0 |
| SiATG3a | +1 | 0 | 0 | -1 | 0 | +1 | +1 | 0 | 0 | -2 | -1 | 0 | +1 | -1 | 0 | 0 | 0 |
| SiATG3b | +1 | 0 | +1 | 0 | +1 | -2 | +2 | 0 | +1 | -2 | -2 | 0 | +3 | -1 | +2 | 0 | 0 |
| SiATG4 | 0 | 0 | +1 | 0 | 0 | -1 | +1 | 0 | 0 | +1 | +1 | 0 | 0 | 0 | 0 | 0 | 0 |
| SiATG5 | +2 | 0 | +2 | 0 | 0 | 0 | +1 | 0 | 0 | +1 | 0 | 0 | +1 | -2 | +2 | 0 | 0 |
| SiATG6a | +1 | +1 | +1 | +1 | 0 | 0 | +1 | +1 | 0 | +2 | +2 | +2 | +3 | -2 | +3 | 0 | +1 |
| SiATG6b | +2 | 0 | 0 | 0 | 0 | 0 | +1 | 0 | 0 | 0 | -1 | 0 | +1 | +1 | 0 | 0 | 0 |
| SiATG7a | +1 | 0 | +1 | -1 | +1 | +1 | +2 | -1 | 0 | -6 | -7 | 0 | +2 | -2 | 0 | -1 | 0 |
| SiATG7b | +2 | 0 | +1 | 0 | 0 | -1 | +2 | +1 | 0 | +1 | +2 | 0 | +2 | -1 | +3 | 0 | 0 |
| SiATG8a | +2 | 0 | +2 | -1 | 0 | -1 | +1 | -1 | 0 | 0 | 0 | 0 | +2 | -1 | 0 | -1 | 0 |
| SiATG8b | +2 | 0 | +2 | 0 | +1 | -6 | +2 | 0 | 0 | -1 | 0 | 0 | +2 | 0 | +2 | 0 | 0 |
| SiATG8c | +1 | 0 | +2 | 0 | 0 | 0 | +1 | 0 | +1 | +1 | +2 | 0 | +2 | 0 | +1 | 0 | 0 |
| SiATG8d | +2 | +1 | 0 | 0 | 0 | 0 | +1 | 0 | 0 | +1 | 0 | +1 | +1 | +1 | +1 | 0 | 0 |
| SiATG9a | +3 | 0 | +2 | +1 | +1 | 0 | +2 | 0 | 0 | +1 | +1 | +1 | +1 | 0 | +2 | 0 | +1 |
| SiATG9b | +1 | 0 | +2 | +1 | +1 | 0 | +2 | 0 | 0 | +1 | +2 | +1 | +2 | 0 | +2 | 0 | 0 |
| SiATG10 | +1 | 0 | +1 | 0 | 0 | 0 | 0 | 0 | 0 | 0 | +1 | 0 | +1 | 0 | +1 | 0 | 0 |
| SiATG11 | 0 | 0 | +1 | 0 | 0 | -1 | +1 | 0 | 0 | +2 | +3 | 0 | +1 | +1 | 0 | 0 | 0 |
| SiATG12 | 0 | 0 | +2 | 0 | 0 | 0 | +1 | 0 | 0 | -1 | -1 | 0 | +1 | 0 | 0 | 0 | 0 |
| SiATG13a | 0 | 0 | +1 | 0 | 0 | +1 | 0 | 0 | 0 | -1 | -1 | 0 | +1 | 0 | 0 | 0 | 0 |
| SiATG13b | +1 | +1 | +1 | 0 | 0 | -1 | +1 | 0 | 0 | 0 | 0 | 0 | +1 | 0 | +1 | 0 | +1 |
| SiATG13c | +1 | +1 | +1 | 0 | 0 | 0 | +2 | 0 | 0 | 0 | 0 | 0 | +2 | 0 | +2 | 0 | 0 |
| SiATG16 | +1 | 0 | +1 | +1 | 0 | 0 | +1 | 0 | 0 | +1 | +2 | 0 | +1 | +1 | +1 | 0 | 0 |
| SiATG18a | -1 | 0 | -1 | 0 | 0 | -3 | 0 | -1 | 0 | +2 | +2 | 0 | 0 | -2 | 0 | 0 | 0 |
| SiATG18b | 0 | 0 | 0 | -1 | 0 | -2 | 0 | -1 | 0 | +2 | +1 | 0 | +1 | -1 | 0 | 0 | 0 |
| SiATG18c | -1 | 0 | 0 | 0 | 0 | -4 | 0 | 0 | 0 | +1 | +1 | 0 | +1 | -2 | 0 | 0 | 0 |
| SiATG18d | +1 | +1 | +2 | 0 | 0 | 0 | +1 | 0 | 0 | +1 | +1 | 0 | +1 | 0 | +1 | 0 | 0 |
| SiATG18e | +1 | 0 | +2 | +1 | +1 | +1 | +1 | 0 | +1 | -2 | 0 | 0 | +2 | 0 | 0 | 0 | 0 |
| SiATG18f | +1 | 0 | 0 | 0 | 0 | -3 | +1 | 0 | 0 | 0 | +1 | 0 | +1 | -1 | +1 | 0 | 0 |
| SiATG18g | +2 | 0 | 0 | 0 | +1 | -1 | +1 | 0 | 0 | +3 | +3 | 0 | +1 | -1 | +2 | 0 | 0 |
| SiATG27 | +1 | 0 | +1 | +1 | 0 | 0 | +2 | 0 | 0 | +1 | +1 | +1 | +1 | -2 | +1 | 0 | +1 |
| SiVTI12a | +1 | 0 | 0 | 0 | 0 | 0 | 0 | 0 | -1 | 0 | 0 | 0 | 0 | +1 | 0 | -1 | -2 |
| SiVTI12b | 0 | -2 | +3 | 0 | 0 | -1 | 0 | +1 | 0 | -2 | 0 | 0 | 0 | +1 | -2 | 0 | -2 |
| SiVPS15 | +1 | +1 | +2 | +1 | +1 | +1 | +2 | +1 | 0 | 0 | +1 | +1 | +2 | 0 | +1 | +1 | 0 |
| SiVPS34 | 0 | 0 | +1 | 0 | +1 | 0 | +2 | +1 | 0 | 0 | +1 | 0 | +2 | +1 | +1 | 0 | -1 |
| SiNBR1 | +1 | 0 | 0 | 0 | 0 | -1 | +1 | 0 | 0 | +1 | +1 | 0 | 0 | 0 | 0 | 0 | 1 |
| SiTOR | -1 | 0 | 0 | -2 | 0 | 0 | 0 | 0 | 0 | 0 | 0 | 0 | 0 | 0 | 0 | -1 | -1 |

**Additional Table S9.Primers used for the qRT-PCR analysis of the 37 SiATG genes.**

| **For gene** | **Primer F (5'-3')** | **Primer R (5'-3')** |
| --- | --- | --- |
| SiATG1 | TCCAGCATCTGGCACCTCACAAG | GAGAACGCCTCCAAGTGTCTGC |
| SiATG2 | TAGATGTCAAGTGGCGGCTGTATGC | TCCTGAGCAGCAATGGATAGCTTAG |
| SiATG3a | GCAGGTGAAGCAGAAGGTCTACG | TACTCTTCCTCAAGCGAGATGGC |
| SiATG3b | GCCAGGCAATCACCTCTCATGTTG | ACAGGCACCAGAGTGCATCCATG |
| SiATG4 | CTTCTGTTCTCGGGCCTCTG | GATGGCACGATTGACTGCAC |
| SiATG5 | TTCGGAAGGTCGAAGGCAGAAG | TCGCAGACTTGCGGAAGCTG |
| SiATG6a | TCTGGAGCACCCGATTTGAC | TTTAGCGCCTTCGTCCAGTT |
| SiATG6b | CAACGTTCCACCTGACGAGT | TCTTCAGCGCCTTTGTCGAA |
| SiATG7a | CTCACCAGATGCGTGGATCA | TCATCAAGTCAGTGAGGCCG |
| SiATG7b | GACGTGAAAGACTGGGGTGT | CTGCTTGCACCAGCAATCTC |
| SiATG8a | GAGGCTGACCGGATCAGAGA | TCTCGGCACTGAGCTTGATT |
| SiATG8b | GTCGTACGGAAGCGGATCAA | GAACAGCCCGAAGGTGTTCT |
| SiATG8c | TCAGGGACAAGTACCCAGACA | GGTGAGATCAGCAGGGACAA |
| SiATG8d | AGCTAGCCTGATGGGAAGTG | GCAGCCAAATGTCTTCTCGC |
| SiATG9a | GCCTCCTCACAGCAGCTTTA | CGGAGGCTCATGGAAACTGT |
| SiATG9b | CGTGGTAAAGAGAGCAGCGA | GGCGTGATGAAAATGGAGGC |
| SiATG10 | CTGGTGAAGAGGTGGAAGGAGATCG | TCTGCACCGTCAGAACTGTCCTC |
| SiATG11 | GAAGAGGCCCGTACCAACAA | GCTTGCTAAGGAGACAGCCA |
| SiATG12 | CGCGGAGGTGGATCAGAAAG | GGTTTGGCGAAAACGCACT |
| SiATG13a | AAATGCACCAGGACTCAGGG | GCGGGTTTCTCCTCTAGCTC |
| SiATG13b | CTGCGCTCTCCTCATGTGAT | AGATGTCCTACGGGGTGTGA |
| SiATG13c | GGTCGTTCAAGAGGGCAGAT | AAGATAACCGACAGCAGCGT |
| SiATG16 | TTGATGTCACCTCGGTCTGC | AGGATGGTTGGCGTGTCATT |
| SiATG18a | CTACCCATCCAAGCCAAGCA | ACGGGTCGATGGACTTGTTC |
| SiATG18b | ATTGCGCTGTCGCCGAATGTG | AGCTGAAGTAATCCGGCAGAATCC |
| SiATG18c | TCATCAGGCTCCATTAGCTGCAATG | ACAAGAACGTCAGGCAGATCAACAG |
| SiATG18d | AGGAGAGGTGCTGACAGAGCAG | AGGATCTGGAGCAGGCATAGGC |
| SiATG18e | TTGCATGGCACTGACAATGGATGG | CGTGGACCTTCAAGAGTGTGTTGC |
| SiATG18f | ACGGCTATGTATCAATGCCACCTG | AGTGCTTGCACCGTTGCTGAG |
| SiATG18g | TATCTGGACCCCCTCTCACG | TCGTGCGTGAACTGTCTGAA |
| SiATG27 | CTCAGCGAGGACGGGTTTTA | GCACTACACTGGGTTCCACA |
| SiVTI12a | GATGCATTGGGGGTTTCTGC | GCACGATGAGCATGCAAGAG |
| SiVTI12b | AGCAAGAAGATCCTGGCAGC | ACCAGAGCCGTGATGATTCC |
| SiVPS15 | ACGGCAAGCACTGAGAATGA | CGTACAGGTGGGTCGTTCAA |
| SiVPS34 | GGACGCCTCTTCCATGTTGA | CCTGACCGTTCCATCAGCTT |
| SiNBR1 | CCTGTTGACCCAGCCCTTAG | GGCACAGTTGTAGGCAGGA |
| SiTOR | GCAATTATAAGTACAGAATTGA | CCAATGCAAGTATAGTATCA |
| SiActin | GGCAAACAGGGAGAAGATGA | GAGGTTGTCGGTAAGGTCACG |

**Additional Table S10. A list of the primer sequences used for the cloning of SiATG8a and the PCR analysis of SiATG8a in transgenic rice.**

| **F1** | ggatcc ATGGCCAGGAGCTCGTTCAA |
| --- | --- |
| **R1** | gaactc TTAGAACAGCCCGAAGGTGT |
| **F2** | \|  \| TGGCATATGCAGCAGCTATA \| \| --- \| --- \| |
| **R2** | \| GCAACAGGATTCAATCTTAAG \| \| --- \| |
| **F3** | CAACCGCATCAGGGAGAAGT |
| **R3** | GATCCGCTTCCGTACGACAT |

**Additional Table S11. Primers used for the for qRT-PCR analysis of endogenous ATG genes of rice.**

| **For gene** | **Primer F (5'-3')** | **Primer R (5'-3')** |
| --- | --- | --- |
| OsATG1a | CCAAACGAGGGCGTGTAAGA | CACCGAGCTCACGTTACAGT |
| OsATG1c | TGCTCCTGCACACGGTTATT | TCCATAGGGGGACATGAGCA |
| OsATG3a | AGCCAAGATCATGCACGGAA | ACAGCAGCATGTTTGCATGG |
| OsATG3b | CTGCCCCTCTCTTGCACAAT | CCAAATACAGGCATGCGACC |
| OsATG4a | GCCGTGACAAAGGTGAACTG | CTCAGAGGCCCGAGAACAGA |
| OsATG4b | GCACTGTCTCGATACCCCAG | CGCTGTAACATGGTAACAGAACG |
| OsATG5 | CAGCTCGAAGGCATCACGTA | TGCCTTGCACCCTTACTAGC |
| OsATG6a | GTGACAAGGTAGGGAGCCAC | GATTGCGCTGCACACAAAGA |
| OsATG6b | TGCAGCTCTGGTTAGGTGAG | TCTCAAGTATGTGTTGCTGAAGAC |
| OsATG8a | ATAGCATGTCCCTGTGCTGG | CGCCCAATCACACTTCAGAC |
| OsATG8b | ACAGTGGCGAGAACACCTTT | ACACATTCACCAGAGCCACA |
| OsATG8c | AAAGCACACGGTGCTCTTAGTA | GCACAGAACACACAAGTATGGAT |
| OsATG8d | AGAAATGGAGAAGCGCAAGCTA | TAGAGGAAGCCATCGCCCTC |
| OsATG8e | CCTCCGTGGAGATCTGCTTT | GGTGGGATAATTAAGTTCGGTATG |
| OsATG9b | ATGGCGATGAAACGTCGGAT | CTGTCCTTCTGAGGGGCATC |
| OsATG12 | AGTCCAAGTTCAAGATTGGTGGA | GTTGCCCGTCGATTCCAAAG |
| OsATG13a | GTCAACCGGCGCTTGTTATG | ATCCGGTTGGTTTTGCTTGC |
| OsATG16 | GCCGGCATGCTGACAAATAG | ATCGCGAGCTAGAGCACATT |
| OsActin | ACCCTGGCTGACTACAACATC | AGTTGACAGCCCTAGGGTG |
